# Deletion of ESX-3 and ESX-4 secretion systems in *Mycobacterium abscessus* results in highly impaired pathogenicity

**DOI:** 10.1038/s42003-025-07572-4

**Published:** 2025-02-03

**Authors:** Wassim Daher, Vincent Le Moigne, Yara Tasrini, Shweta Parmar, Danielle L. Sexton, John Jairo Aguilera-Correa, Valentin Berdal, Elitza I. Tocheva, Jean-Louis Herrmann, Laurent Kremer

**Affiliations:** 1 Centre National de la Recherche Scientifique UMR 9004, Institut de Recherche en Infectiologie de Montpellier (IRIM), Université de Montpellier, 1919 route de Mende, 34293 Montpellier, France; 2 INSERM, IRIM, 34293 Montpellier, France; 3 Université Paris-Saclay, UVSQ, Inserm, Infection et inflammation, 78180 Montigny-Le-Bretonneux, France; 4 Department of Microbiology and Immunology, University of British Columbia, Vancouver, Canada

**Keywords:** Bacteriology, Infection

## Abstract

Type VII secretion systems participate in protein export, virulence, conjugation, and metabolic regulation. Five subtypes (ESX-1 to ESX-5) exist, each with specific roles and well-characterized secretion profiles in various mycobacterial species. *Mycobacterium abscessus*, encodes only ESX-3 and ESX-4. Here, single and double *M. abscessus* mutants lacking the main ATPases EccC3 and EccC4 were used to define ESX-3 and ESX-4 contributions to substrate secretion and virulence. Our results demonstrate that EsxG/H secretion depends entirely on ESX-3, whereas both ESX-3 and ESX-4 secrete EsxU/T. Furthermore, two newly identified PE/PPE substrates (MAB_0046/MAB_0047) require ESX-3 for secretion. Functional complementation restored secretion and revealed subpolar localization of these systems. Macrophage infections showed that ESX-3 and ESX-4 contribute to bacterial internalization, phagosomal escape, and intracellular survival. In mice, infections with *eccC3-* and/or *eccC4*-deletion mutants resulted in complete survival and reduced bacterial loads in the lungs. These findings demonstrate that both ESX systems drive *M. abscessus* pathogenicity.

## Abstract (web summary)

Investigation of ESX-3 and ESX-4 secretion systems in *Mycobacterium abscessus* reveals their crucial role in substrate secretion, macrophage infection, and in vivo pathogenicity, highlighting their role in mycobacterial survival and host interactions.

## Introduction

Pathogenic bacteria, including mycobacteria, have evolved specialized secretion machineries to export virulence factors, such as effector proteins, to enhance their survival within hosts. To secrete a diverse range of effectors, mycobacteria rely on the Type VII Secretion System (T7SS), also named the ESAT-6 secretion system (ESX)^1^. In *Mycobacterium tuberculosis* (*Mtb*), the T7SS systems are encoded on five genetic loci (ESX-1 to ESX-5) and are involved in various roles during growth and pathogenesis^2–8^. For example, ESX-1 secretes the key effectors EsxA and EsxB required for intracellular survival in macrophages, ESX-3 is involved in iron and zinc acquisition as well as modulation of host immunity, and ESX-5, found uniquely in slow-growing and pathogenic species, is involved in immune modulation and nutrient uptake^9^. An indirect role has been suggested for ESX-4 during DNA conjugation in *Mycobacterium smegmatis* (*Msmeg*)^8^. *Mycobacterium abscessus (Mab)* encodes two of the five ESX systems, which facilitates the investigation of the roles of ESX-3 and ESX-4 in this species^10^. Notably, the ESX systems share conserved core components (EccA, EccB, EccC, EccD, EccE and the protease MycP) that form a channel across the inner membrane (IM) to facilitate effector transport^11^.

*Mab* is a rapidly-growing nontuberculous mycobacterium (NTM) that has significantly contributed to the global rise of NTM infections^12,13^. It is the causative agent of lung diseases in individuals with cystic fibrosis (CF) and recent studies have documented the rising incidence of pulmonary NTM infections in the aging population^14^. Managing *Mab* infections is challenging due to the innate and acquired antibiotic resistance in this bacterium^15^. Deletion studies of ESX-3 in *Mtb* and *Mab* have previously shown that the system is essential for pathogenesis^16^. Under standard laboratory conditions, *Mtb* relies on the ESX-3 system for growth, whereas *Mab* does not^16,17^. Recent studies have also revealed a functional link between ESX-3 and iron uptake in *Mab* and have shown that interfering with ESX-3 function results in the production of an unusual mycobactin siderophore variant with cytotoxic properties^18^. These findings deepen our understanding of the role of ESX-3 in iron homeostasis and highlight key differences in iron-acquisition strategies between *Mab* and *Mtb*. While deletion of the complete e*sx-3* locus in *Mab* mutant exhibited impaired survival in macrophages and less pathology in mice, the role of *esx-3* in *Mab* virulence has not been confirmed by complementing studies^16^.

Given the limited understanding of the virulence-related function of ESX-4 in other mycobacterial species, it has been hypothesized that the activity of ESX-4 in *Mab* and the *M. chelonae-abscessus* complex may depend on the presence of a functional EccE4 component, which is absent from other mycobacteria^10^. To get insights into the mechanisms of pathogenesis, a transposon library screen of a clinical *Mab* strain identified essential genes for growth in amoeba and macrophages, underscoring the critical role of the *esx-4* locus by revealing 12 distinct transposon insertions^19^. Moreover, the deletion of *eccB4*, a core component of the ESX-4 secretion system, led to accelerated phagosomal acidification and impaired disruption of the phagosomal membrane in infected macrophages, key strategies employed by *Mab* for intracellular survival^19^. This mutant also exhibited impaired secretion of *esx4*-encoded effector proteins EsxU/T, confirming the role of ESX-4 as a functional secretion apparatus in *Mab*^19^.

To investigate the specific roles of ESX-3 and -4 in effector secretion and *Mab* pathogenesis, here we generated single (Δ*eccC3* and Δ*eccC4*) and double (Δ*eccC3/*Δ*eccC4*) deletion mutants, characterized their phenotypes, and determined their sub-cellular localization and secretion profiles. Next, we examined the effects of these deletions on growth in planktonic cultures and biofilms (in vitro), as well as assessed their survival in macrophage (ex vivo) and mouse (in vivo) models. Our findings reveal that while the strain lacking both systems can grow in vitro, ESX-3 and ESX-4 are required for survival in macrophages and for pathogenesis in the mouse host.

## Results

### Characterization of single and double *eccC* deletion mutants

**Fig. 1:**
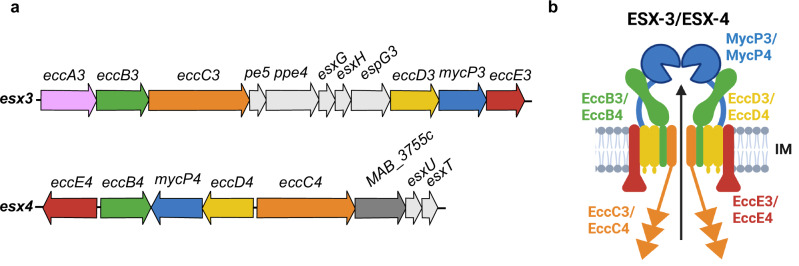
The ESX-3 and ESX-4 secretion systems in *M. abscessus.* **a** Schematic representation of the genetic organization of the *esx-3* and *esx-4* loci. The genes encoding the different ESX core components are shown using different colors. Genes encoding the secreted substrates are in gray. *MAB_3755c* (dark gray) encodes a protein with unknown function. **b** Model of the ESX-3/4 core components using the same color code than in (**a**). The ATPase domains of EccC3/EccC4 are indicated with triangles.

Unlike most mycobacterial species, *Mab* only possesses ESX-3 and ESX-4 secretion systems **(**Fig. 1**)**^20^. Among the ESX core components, EccC is an ATPase, providing the energy for secretion^21^. The Δ*eccC3* and Δ*eccC4* mutants were generated in both the glycopeptidolipid (GPL)-producing smooth (S) and GPL-deficient rough (R) variants of *Mab* using an unmarked deletion method **(**Supplementary Fig. 1a**)**^22,23^. Genotypes of the parental and mutant strains were confirmed with PCR using the primers in Supplementary Table 1**(**Supplementary Fig. 1b**)**. Genetic complementation of Δ*eccC3* and Δ*eccC4* was done by integrating *eccC3* or *eccC4* fused with a C-terminal HA-tag, under the control of the *hsp60* promoter^24^.

The single and double Δ*eccC3* and Δ*eccCC4* mutants displayed normal growth in standard 7H9 broth compared to their respective *Mab* S and R parental strains **(**Supplementary Fig. 2a-2b**)**. However, in iron-limited GAST medium, the Δ*eccC3* and Δ*eccC3/*Δ*eccC*4 mutants in the S background exhibited a 37% reduction in growth rate (*P* < 0.0001), and a 41% reduction in the R background (*P* < 0.0001) compared to the corresponding wild-type strain (WT), underscoring the necessity of ESX-3, but not ESX-4, for optimal growth under low-iron conditions **(**Supplementary Fig. 2a–2c**)**^18^. For biofilm development, we monitored the growth and appearance of colony-biofilms grown on a Whatman polycarbonate nuclepore filter discs (0.2 µm)^25^. Deletion of both ESX systems did not alter the characteristics of the S mutant; however, Δ*eccC3* and Δ*eccC3/*Δ*eccC4* mutants exhibited orange pigmentation due to the extracellular accumulation of carboxymycobactin-Fe^3+^ and the intracellular accumulation of mycobactin-Fe^3+^ complexes^26^, indicative of impaired ESX-3^18^ **(**Supplementary Fig. 2d, left panel**)**. These mutants also showed a striking 99% reduction in CFU counts in biofilms (*P* = 0.0011) compared to the parental S strain **(**Supplementary Fig. 2d, right panel). Complementation of Δ*eccC3* restored both the lack of pigmentation and bacterial loads, indicating that the deletion of *eccC3* significantly impaired biofilm viability. In contrast, *eccC4* deletion did not affect biofilm formation (Supplementary Fig. 2d). Similar biofilm results were observed in the R variant (Supplementary Fig. 2e). Of note, although the aspect of the colony-biofilms appeared similar between the strains, the number of viable mycobacteria was significantly reduced in the Δ*eccC3* and Δ*eccC3/*Δ*eccC4* compared to the control strains, which may be explained by differences in bacterial density. We further tested the susceptibility of the mutants to H_2_O_2_ using the disk diffusion method (Supplementary Fig. 3). RΔ*eccC3* and RΔ*eccC3/*Δ*eccC4* were slightly more sensitive than RΔ*eccC4* to H_2_O_2_ when compared to the parental strain, and complementation of the single mutants restored the WT phenotype, thus indicating that both T7SS contribute to *Mab* adaptation to oxidative stress.

### Deletion of *eccC3* and *eccC4* results in non-functional ESX-3 and ESX-4 systems

**Fig. 2:**
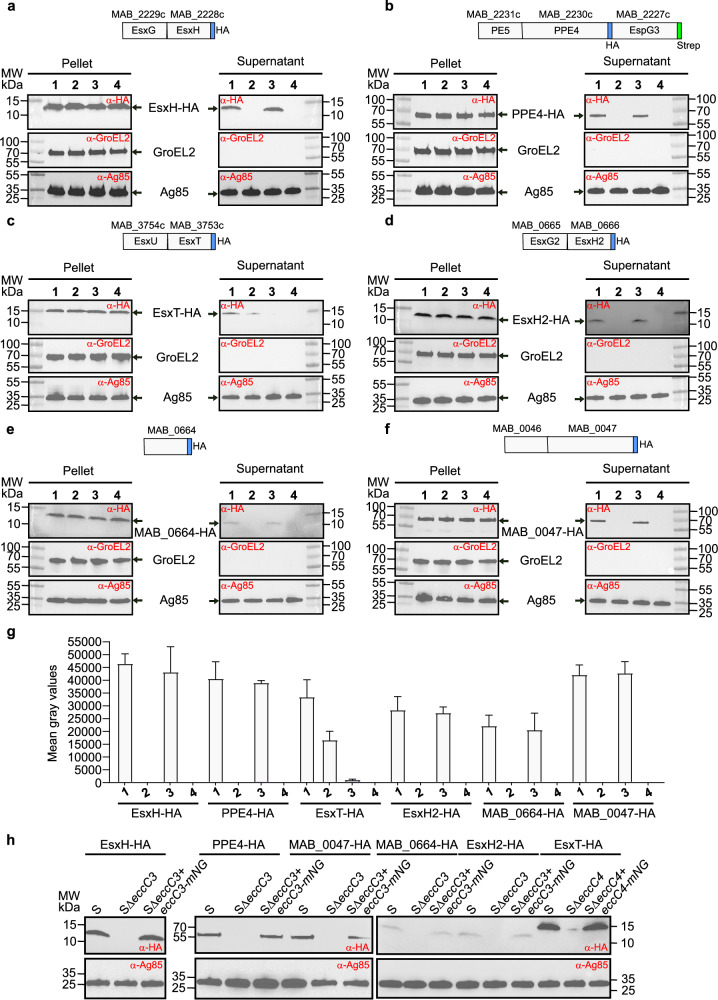
Analysis of secretion proteins in *M. abscessus* lacking ESX-3 and/or ESX-4. Immunoblot analysis of bacterial total lysates (Pellet) and secreted proteins (Supernatant) in *Mab* S (lane 1), Δ*eccC3* (lane 2), Δ*eccC4* (lane 3), and Δ*eccC3/*Δ*eccC4* (lane 4). Immunoblots illustrating the secretion profile of EsxH (**a**), PPE4 (**b**), EsxT (**c**), EsxH2 (**d**), MAB_0664 (**e**), and MAB_0047 (**f**). Anti-Ag85 antibodies were used as loading controls to verify that the secretion of Ag85 is not affected by the disruption of ESX-3 or ESX-4. GroEL2 serves as a loading control for total lysates and to validate the absence of bacterial lysis. This figure presents data from experimental replicate 1 only. The secreted proteins were tagged with HA tag and expressed from integrative plasmids. Specific proteins were identified using anti-HA antibodies. The design of the fusion proteins and their tags is shown above each blot: (**a**) EsxG/EsxH-HA, (**b**) PE5-PPE4-HA-EspG3-STREP, (**c**) EsxU/EsxT-HA, (**d**) EsxG2/EsxH2-HA, (**e**) MAB_0664-HA, (**f**) MAB_0046-MAB_0047-HA. **g** Quantification of the bands present in the supernatant fractions shown in panels (**a–f**) using the Fiji software (mean gray values) (n = 3). **h** Secretion analysis of the same substrates in complemented strains expressing either EccC3-mNeonGreen or EccC4-mNeonGreen. Data are mean ± SD.

ESX secretion profiles have been investigated in various mycobacteria. While most substrates are secreted by specific ESX systems, exceptions like CpnT^27^ show the involvement of multiple ESX systems in substrate secretion. Additionally, studies in *M. marinum* indicate that disruptions in one system can indirectly affect the activity of the others^28^. We reasoned that the presence of only ESX-3 and ESX-4 in *Mab* offers a simplified model that could offer unique insights into the interactions between these two systems and their respective contributions to mycobacterial physiology. By generating single and double deletion mutants, we aimed to eliminate background activities and overlapping functions during protein secretion. To investigate which ESX system facilitates the secretion of specific effectors within the *esx* loci of *Mab* (*esxG/esxH* and *pe5/ppe4* for ESX-3, *esxU/esxT* for ESX-4; Fig. 1a), effector genes were cloned under the constitutive *hsp60* promoter into an integrative vector, fused with an HA-tag, and introduced into various *Mab* strains. To explore the role of ESX-3 and ESX-4 in the secretion of these effectors, we examined total bacterial lysates and culture supernatants from Δ*eccC3*, Δ*eccC4*, and Δ*eccC3/*Δ*eccC4*. The results provide critical insights into the substrate specificity of these ESX systems. Western blot analysis of EsxH-HA (Fig. 2a and Supplementary Fig. 4a) revealed its secretion in WT and Δ*eccC4* strains but not in Δ*eccC3* or Δ*eccC3/*Δ*eccC4*, unequivocally demonstrating that EsxH is secreted exclusively *via* the ESX-3. Similarly, the PE5/PPE4-HA pair (Fig. 2b and Supplementary Fig. 4b) was secreted only in strains where EccC3 was functional. Interestingly, secretion of EsxT-HA was partially reduced in Δ*eccC3* but strongly reduced in Δ*eccC4* and abrogated in Δ*eccC3/*Δ*eccC4* (Fig. 2c and Supplementary Fig. 4c). These results indicated that EsxT secretion was facilitated by both ESX-3 and ESX-4. Notably, ESX-4 played a critical role in EsxT translocation. We next examined substrates encoded outside the canonical *esx* loci (Supplementary Fig. 5a-5b). We identified a second EsxG2/H2 substrate pair (MAB_0665/MAB_0666), differing from EsxG/EsxH by one amino-acid substitution in EsxG2 (99% identity with EsxG) (Supplementary Fig. 5c). EsxH2-HA (Fig. 2d and Supplementary Fig. 4d), encoded within the *MAB_0665/0666* locus, was exclusively secreted *via* ESX-3, as evidenced by its absence in Δ*eccC3* and Δ*eccC3/*Δ*eccC4*. Adjacent to this pair is *MAB_0664* (encoding a protein sharing 65% identity with PE5 from *Mtb*) (Supplementary Fig. 5d). Similarly, MAB_0664-HA (Fig. 2e and Supplementary Fig. 4e) secretion was strictly dependent on ESX-3. Additionally, the *MAB_0046/MAB_0047* locus, encoding proteins sharing 75% and 46% identity with PE5 and PPE4 from *Mtb*, respectively, was also analyzed (Supplementary Fig. 5e). The MAB_0046/MAB_0047-HA locus (Fig. 2f and Supplementary Fig. 4f) was secreted exclusively by the ESX-3, as secretion was observed in the WT and Δ*eccC4* strains but not in Δ*eccC3* or Δ*eccC3/*Δ*eccC4*. This indicated that both loci were dependent on ESX-3 for their translocation. These observations were further supported by the use of Ag85 as a control for secretion, which was unaffected by the deletion of *eccC3* or *eccC4*, underscoring the specificity of the secretion defects for ESX-3 and ESX-4 substrates. Additionally, the absence of GroEL2 in culture supernatants excluded the presence of cytoplasmic components due to bacterial lysis or artifactual protein detection in the supernatants. Quantitative analysis of secretion patterns corroborated these results (Fig. 2g), and revealed a clear reduction or absence of secretion in mutants lacking the corresponding ESX machinery. Furthermore, complementation of Δ*eccC3* and Δ*eccC4* with *eccC3-mNeonGreen* or *eccC4-mNeonGreen*, respectively, restored substrate secretion to levels comparable to the WT strain (Fig. 2h). Complementation was achieved by expressing C-terminally mNeonGreen-tagged versions of these proteins under the constitutive *hsp60* promoter from an integrative vector (Supplementary Tables 2 and 3).

In summary, ESX-3 is required for the secretion of EsxH, PPE4, EsxH2, MAB_0664, and MAB_0047, while ESX-4 contributes uniquely to the secretion of EsxT, reflecting a sophisticated interplay between these ESX systems in substrate translocation in *Mab*.

### ESX-3 and ESX-4 localize to the subpolar regions of *Mab*

**Fig. 3:**
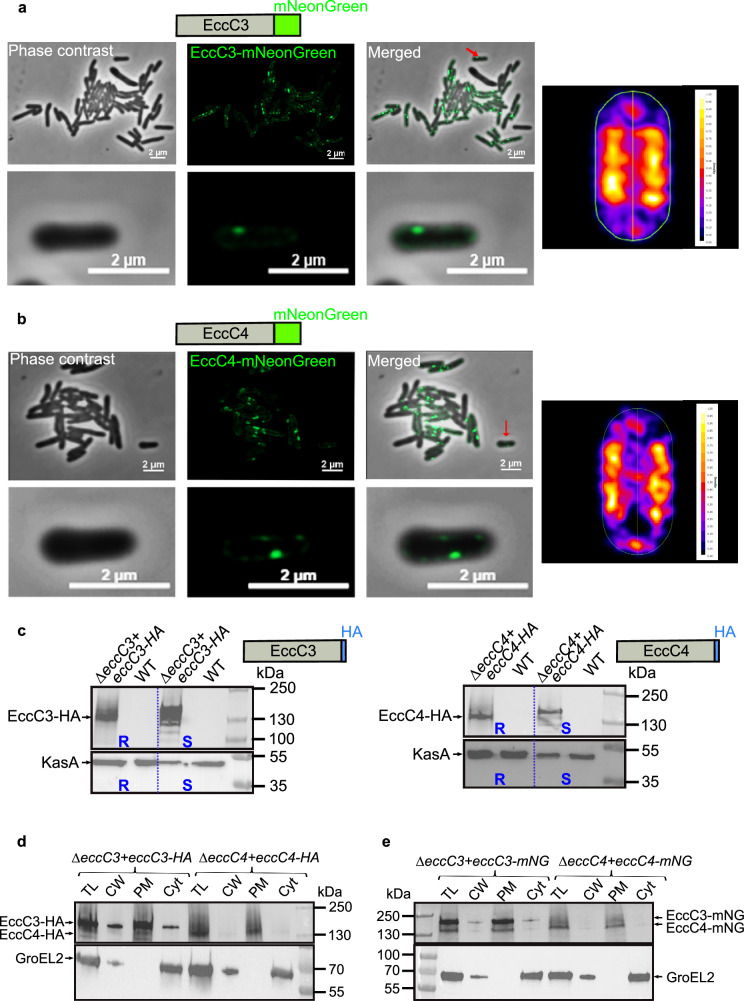
Subcellular localization of ESX-3 and ESX-4 in *M. abscessus.* Immunofluorescence microscopy of *Mab* Δ*eccC3* and Δ*eccC4* mutants producing EccC3 (**a**) or EccC4 (**b**) fused to mNeonGreen. Insets represent individual bacilli. Heat maps indicating the localization patterns of fluorescent foci in 50–100 *Mab* cells are provided. **c** Detection of EccC3-HA and EccC4-HA in crude lysates of *Mab* R and S lacking either *eccC3* (Δ*eccC3*) or *eccC4* (Δ*eccC4*) carrying the *eccC3-HA* and *eccC4-HA* constructs. The genes were under the control of the *hsp60* promoter and integrated into the genome. Proteins were detected using Western blot and anti-HA antibodies (upper panel) or anti-KasA antibodies (lower panel, included as a loading control). Positions of EccC3-HA and EccC4-HA are indicated. Lysates of Δ*eccC3* and Δ*eccC4* mutants complemented with either EccC3-HA or EccC4-HA (**d**) and EccC3-mNeonGreen or EccC4-mNeonGreen (**e**) were subjected to sub-cellular fractionation. The fractions were probed with anti-HA antibodies or anti-mNeonGreen antibodies to detect the respective fusion proteins, while anti-GroEL2 antibodies were used as a cytosolic marker. TL, total lysate; CW, cell wall; PM, plasma membrane; Cyt, cytosol. Positions of EccC3-HA or EccC3-mNeonGreen and EccC4-HA or EccC4-mNeonGreen are indicated.

To explore the sub-cellular localization of EccC3 and EccC4 in *Mab*, the mNeonGreen-tagged versions of these proteins were introduced into Δ*eccC3* and Δ*eccC4* strains to avoid interference with native EccC components. In contrast to previous studies showing polar localization of the T7SS in *Msmeg*^29,30^, *Mab* strains expressing EccC3-mNeonGreen and EccC4-mNeonGreen exhibited a distinct subpolar localization at the membrane (Fig. 3a, b). This pattern was consistently observed in live cells and validated with heat maps using the MicrobeJ plugin in ImageJ (Fig. 3a, b)^31^.

To further validate membrane association of EccC3 and EccC4, sub-cellular fractionation combined with Western blotting was performed using Δ*eccC3* and Δ*eccC4* strains complemented with HA-tagged versions of *eccC3* and *eccC4*, respectively. Figure 3c shows the presence of EccC3-HA (left panels) and EccC4-HA (right panels) in their respective recombinant strains, while no bands were detected in the parental S and R control strains. The protein KasA, used as a loading control, was detected in all samples. Fractionation studies on Δ*eccC4* producing EccC4-HA revealed its presence in the plasma membrane, with minimal detection in the cell wall and cytosol, unlike the cytoplasmic marker GroEL2 (Fig. 3d)^27,32^. Similarly, when EccC4-mNeonGreen was expressed in Δ*eccC4*, the protein localized mainly in the plasma membrane fraction (Fig. 3e). In the case of Δ*eccC3* complemented with EccC3-HA, fractionation studies demonstrated that EccC3-HA was predominantly found in the plasma membrane, with lower amounts detected in the cell wall and cytosol, likely attributable to fraction contamination (Fig. 3d). A comparable distribution was observed in Δ*eccC3* complemented with EccC3-mNeonGreen, further substantiating the plasma membrane localization of EccC3 and validating the consistency of these results across the different detection approaches (Fig. 3e).

Together, super-resolution fluorescent light microscopy and Western blot analysis of sub-cellular fractions confirmed that EccC3 and EccC4 localized to the subpolar regions of *Mab* and were membrane-associated components.

### Distinct survival rates of Δ*eccC3* and Δ*eccC4* mutants in macrophages

**Fig. 4:**
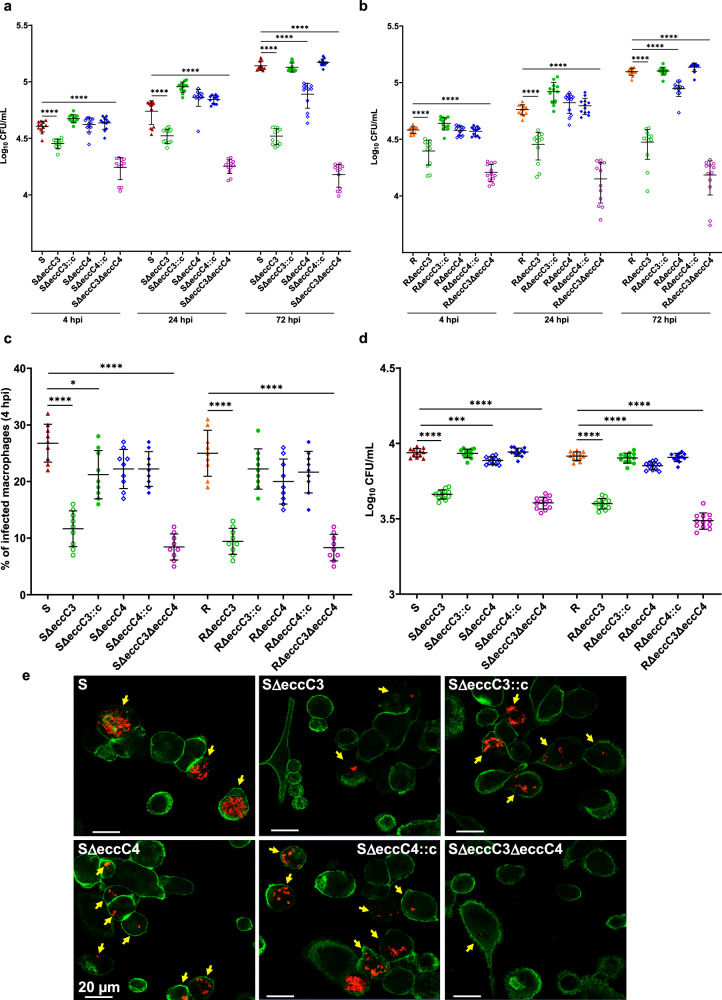
EccC3 is crucial for the uptake and intracellular replication of *M. abscessus.* **a** Infection assays with THP-1 macrophages using fluorescent *Mab* S-derived strains (MOI of 2:1). CFU were determined at 4, 24, and 72 hpi. The data are presented as mean values from three independent experiments (n = 12). Statistical significance was determined using Tukey’s multiple comparisons test: ****, P < 0.0001. **b** THP-1 cells were infected with the different fluorescent *Mab* R (MOI of 2:1). CFU counts were similarly determined at 4, 24, and 72 hpi. The data are presented as mean values from three independent experiments (n = 12). Statistical significance was determined using Tukey’s multiple comparisons test: ****, *P* < 0.0001. **c** Percentage of infected macrophages was determined for each of the S- or R-derived mutants and their complemented strains. Data represent mean values from three independent experiments, each performed in triplicate (n = 9). Statistical significance was determined using Tukey’s multiple comparisons test: *, *P* < 0.05, ****, *P* < 0.0001. **d** Adhesion assays to evaluate the binding of *eccC* mutant strains to THP-1 macrophages. Macrophages were pre-cooled to prevent bacterial internalization and exposed to the bacterial strains at a MOI of 100 for 1 hr at 4 °C to enhance bacteria-cell contact. CFUs were quantified to assess adhesion. The data reflect mean values from three independent experiments, each in quadruplicate (n = 12). Statistical analysis was assessed using the one-tailed Mann-Whitney test: ***, *P* < 0.001, ****, *P* < 0.0001. **e** Immunofluorescence microscopy images were taken after 72 hpi, using a confocal microscope at 40x magnification, show macrophages (stained green) infected with various *Mab* strains (red). Yellow arrows point to mycobacteria-infected cells. Data are mean ± SD.

To explore the intracellular survival rates of ESX-3 and ESX-4 deletion strains, bacterial load at different time points was monitored in human THP-1 macrophages infected with WT S and R *Mab*, SΔ*eccC3*, SΔ*eccC4*, RΔ*eccC3*, RΔ*eccC4* and their corresponding complemented strains, and with SΔ*eccC3/*Δ*eccC4* and RΔ*eccC3/*Δ*eccC4*. As revealed by CFU counts, both morphotypes exhibited impaired growth of the Δ*eccC3* and Δ*eccC3/*Δ*eccC4* compared to the parental and complemented strains shortly after infection (4 hpi; Fig. 4a, b). This defect persisted and intensified at 24 and 72 hpi, indicating a significantly lower intracellular bacterial load in Δ*eccC3* and Δ*eccC3/*Δ*eccC4*, with the latter showing a more pronounced defect. Complementation with *eccC3* restored normal intracellular growth, as shown by fluorescence microscopy at 72 hpi (Fig. 4e and Supplementary Fig. 6a). The early defect in intracellular survival of SΔ*eccC3* and SΔ*eccC3/*Δ*eccC4* at 4 hpi pointed to perturbed bacterial internalization. Consistent with these findings, adhesion assays revealed a marked decrease in cell adhesion and bacterial entry for Δ*eccC3* and Δ*eccC3/*Δ*eccC4*, unlike the parental or the Δ*eccC3* complemented strains (Fig. 4d, Supplementary Fig. 6b, c). In contrast, *eccC4* deletion had minimal impact on bacterial uptake by macrophages (Fig. 4d), but it resulted in reduced growth at 72 hpi (Fig. 4a, e). Quantifying the percentage of infected macrophages at 4 hpi emphasized a reduced proportion of cells harboring Δ*eccC3* or Δ*eccC3/*Δ*eccC4* compared to those infected with S/R or their corresponding Δ*eccC4* mutants (Fig. 4c), consistent with the CFU results (Fig. 4d). Overall, these findings indicated that Δ*eccC3* and Δ*eccC4* could not compensate for each other in macrophages. Both ESX systems contribute differently to the infection process in macrophages, while sharing similar roles in the S and R *Mab* variants.

### ESX-3 and ESX-4 are required for blocking phagosome maturation and rupturing phagosomal membranes

**Fig. 5:**
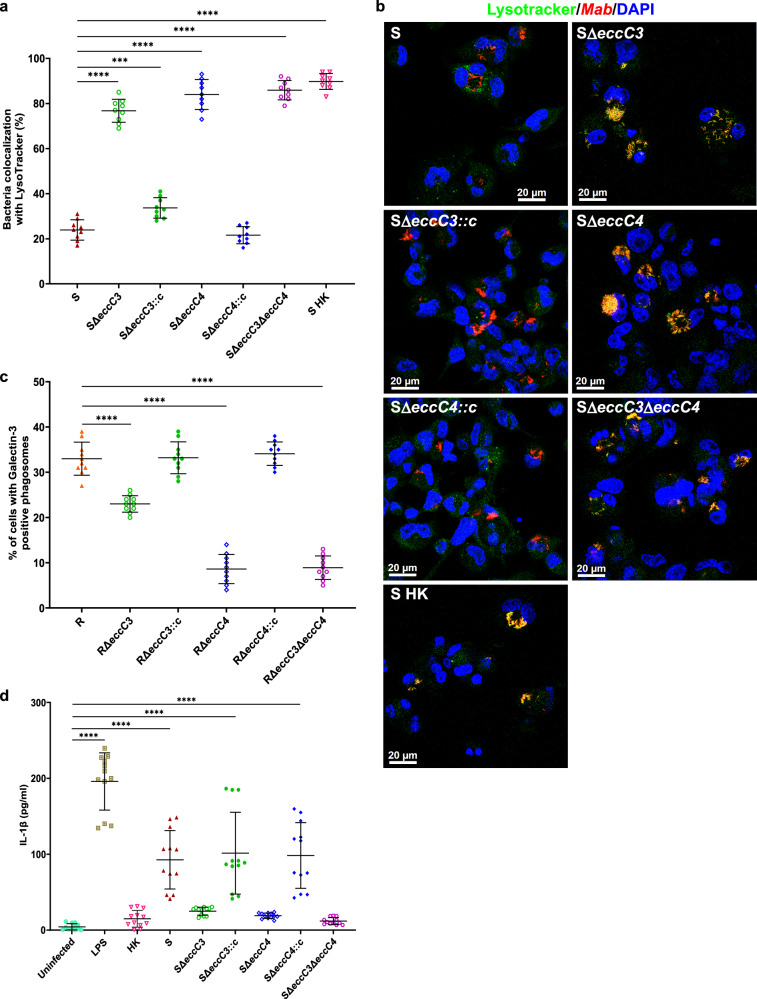
Both ESX-3 an ESX-4 are required for blocking phagosome maturation and phagosomal escape. **a** Co-localization of *Mab* S, SΔ*eccC3*, SΔ*eccC3::c*, SΔ*eccC4*, SΔ*eccC4::c*, SΔ*eccC3/*Δ*eccC4*, and heat-killed strains (red) with the acidotropic dye LysoTracker (green) in infected THP-1 cells at 20 hpi. Data are presented as means from three independent experiments (n = 9). Statistical significance was assessed using Tukey’s test: ***, *P* <  0.0001, ****, *P* < 0.00001. **b** Acidic compartments were visualized by confocal microscopy using LysoTracker, a fluorescent marker for acidic compartments, in infected macrophages. Co-localization of red fluorescent signals from *Mab* S, SΔ*eccC3*, SΔ*eccC3::c*, SΔ*eccC4*, SΔ*eccC4::c*, SΔ*eccC3/*Δ*eccC4*, and heat-killed bacteria with LysoTracker was observed in infected THP-1 cells. Yellow coloring indicated co-localization of red and green labeling. **c** Percentage of infected macrophages containing at least one positively-stained phagosome (Gal-3^+^). The values represent the mean of 1000 infected cells analyzed from three different experiments (n = 10). *P* values were determined by ANOVA with Tukey’s test; ****, *P* < 0.0001. **d** IL-1β production during THP-1 infection by *Mab* S, SΔ*eccC3*, SΔ*eccC3::c*, SΔ*eccC4*, SΔ*eccC4::c*, SΔ*eccC3/*Δ*eccC4*, and heat-killed strains. The data reflect mean values from three independent experiments, each in quadruplicate (n = 12). *P* values were determined by ANOVA with Tukey’s test; ****, *P* < 0.0001. Data are mean ± SD.

To survive and replicate, pathogenic mycobacteria, such as *Mtb*, prevent phagosome maturation by preventing fusion with lysosomes^33^. Using LysoTracker Green to track the acidified compartments, we investigated whether the impaired intracellular growth of Δ*eccC3* and Δ*eccC4* was linked to lysosomal degradation. While the WT strain showed only ~25% co-localization with acidic compartments, Δ*eccC3*, Δ*eccC4*, and Δ*eccC3/*Δ*eccC4* exhibited much higher co-localization (~80%), similar to heat-killed bacteria (Fig. 5a, b). Complemented strains resembled the parental strain in their low co-localization rates, indicating that both ESX-3 and ESX-4 were crucial in avoiding lysosomal degradation (Fig. 5a, b). We also examined the co-localization of the strains with FK2, a marker for ubiquitinated proteins. Δ*eccC3* and Δ*eccC3/*Δ*eccC4* co-localized with FK2, unlike the other strains (Supplementary Fig. 7a-7b). Thus, in the absence of ESX-3, the bacilli were more likely to be tagged for ubiquitination, potentially labeling the pathogen for degradation *via* the proteasomal or autophagy host pathways. This suggested a potential role for ESX-3 in evading host immune mechanisms that target intracellular pathogens for destruction. A possible explanation could be that ESX-3 prevented *Mab* from the ubiquitin-mediated degradation processes, thus favoring the establishment of its intracellular niche.

Previous studies have shown that *Mab*, like *Mtb*, can damage phagosomal membranes, exposing them to host autophagic pathways^34^. Infected macrophages with damaged phagosomes were tagged by galectin-8, directing bacteria to selective autophagy^35^. Using fluorescently-labeled galectin-3 to mark ruptured phagosomal membranes, we observed relatively high percentage of galectin-3 positive phagosomes in cells infected with WT *Mab*, while Δ*eccC3* and Δ*eccC4* mutants showed lower galectin-3 positivity (Fig. 5c and Supplementary Fig. 8). Complemented strains restored the WT levels of galectin-3 labeling, suggesting that both ESX-3 and ESX-4 contribute to phagosomal membrane damage (Fig. 5c). We further confirmed the role of ESX-3 and ESX-4 in promoting phagosome-to-cytosol communication by measuring IL-1β production, a marker of inflammasome activation. High levels of IL-1β were secreted by macrophages infected with wild-type *Mab* and the complemented strains, whereas the single and double mutants produced significantly lower IL-1β, akin to heat-killed bacteria (Fig. 5d).

Overall, these observations identified critical roles for both ESX systems in facilitating phagosomal membrane rupture and subsequent escape into the cytosol.

### Both *eccC3* and *eccC4* are needed for *Mab* virulence in mice

**Fig. 6:**
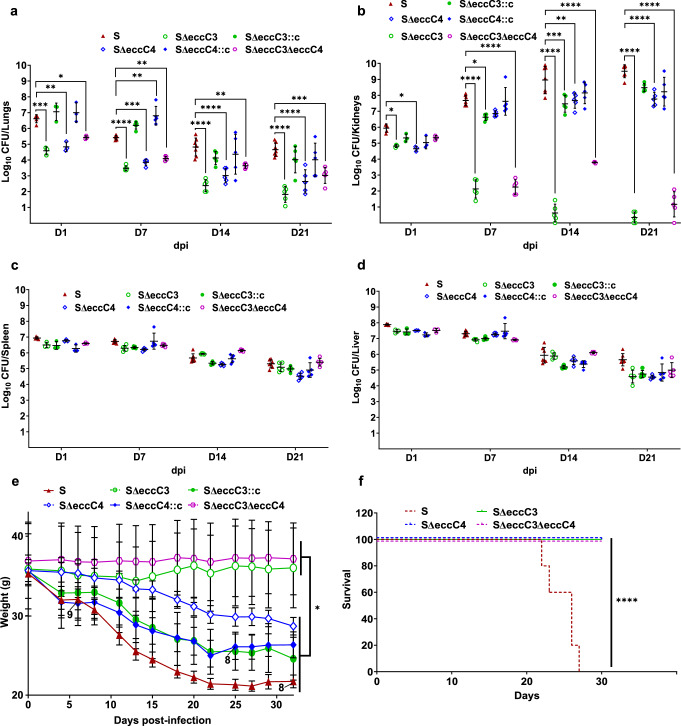
Effect of ESX-3 and/or ESX-4 depletion on *M. abscessus* pathogenicity in mice. C3HeB/FeJ mice were infected intravenously with 2 × 10^7^ CFU/mouse of WT S, SΔ*eccC3*, SΔ*eccC4*, and complemented strains, as well as with SΔ*eccC3/*Δ*eccC4*. At 1, 7, 14, and 21 dpi. Lungs (**a**), kidneys (**b**), spleen (**c**), and liver (**d**) were collected for CFU determination. Five mice were used per group (n = 5 mice) (three for day 1, n = 3 mice). Differences between means were analyzed by unpaired *t* test (**a–d**) using GraphPad Prism. **e** Temporal evolution of mice body weights. Numbers in the graph indicate the number of mice analyzed in the experiment (n = 10 mice). **f** Survival curves of mice infected with WT S, SΔ*eccC3*, SΔ*eccC4*, and SΔ*eccC3/*Δ*eccC4* (n = 5 mice per condition). Results in (**e**) and (**f**) were generated from two independent experiments. Five mice per group (three for day 1) were used, and differences between means were analyzed by two-way ANOVA with Tukey post-test for multiple comparisons (**e**). n.s., non-significant; *, *P* < 0.05; **, *P* < 0.01; ***, *P* < 0.001; ****, *P* < 0.0001. Survival statistics were assessed using the log-rank (Mantel-Cox) test for Kaplan-Meier survival curves (**f**); ****, *P* < 0.0001. Data are mean ± SD.

To further explore the role and contribution of each ESX system with respect to pathogenesis in the context of functional innate and adaptive immunity, C3HeB/FeJ mice were exploited as a persistent infection model for *Mab* S^36,37^. Mice were intravenously infected with either the WT S morphotype or its derived strains (SΔ*eccC3*, SΔ*eccC4*, SΔ*eccC3::c*, SΔ*eccC4::c* and SΔ*eccC3/*Δ*eccC4*). CFU counts were determined in the lungs, kidneys, spleen, and liver at 1, 7, 14, and 21 dpi. At 1 dpi, CFU counts were significantly lower in the lungs of mice infected with the single and double mutants compared to those infected with the S strain, indicating reduced lung colonization. Complementation with *eccC3* or *eccC4* restored bacterial persistence to levels comparable to the parental S strain (Fig. 6a). This reduction in bacterial load persisted through 7, 14, and 21 dpi. In the kidneys, a distinctive pattern emerged. While *Mab* proliferated steadily in these organs (Fig. 6b)^36^, the deletion of *eccC3*, but not *eccC4*, led to a massive drop in CFU counts (also observed in the Δ*eccC3/*Δ*eccC4* mutant), approaching sterilization by 14 dpi (Fig. 6b). In contrast, the spleen (Fig. 6c) and liver (Fig. 6d) did not show significant decrease of CFU counts for the mutants compared to the WT strain, following a similar decline over time. Moreover, mice infected with the single SΔ*eccC3* or double mutants maintained a stable body weight while the WT S and the complemented SΔ*eccC3::c* showed a significant decrease in body weight (Fig. 6e). Mice infected with SΔ*eccC4* exhibited an intermediate phenotype with a delay in body weight decrease starting at 10 days post-infection. Notably, all mice infected with *Mab* S succumbed by 27 dpi, while mice infected with any of the single or double deletion mutants survived up to 30 dpi, the endpoint of the experiment (Fig. 6f). This marked difference indicated that the absence of either *eccC3* or *eccC4* greatly attenuated the virulence of *Mab*, as evidenced by the significantly reduced bacterial loads in different organs and the survival of the infected mice.

## Discussion

In this study, we generated and characterized single and double deletion mutants to elucidate the functional roles and sub-cellular localization of EccC3 and EccC4, key components of the ESX-3 and ESX-4 secretion systems in *Mab*. Since ESX-3 is the most conserved among all T7SS, it has been extensively studied in other mycobacterial species. The *esx-3* genes are activated under iron-limited conditions^38,39^ and encode several substrates, including EsxG and EsxH, PE5, and PPE4. In *Mtb*, the EsxG/H substrate pair disrupts lysosomal trafficking and inhibits phagosome maturation^40,41^, crucial for intracellular growth and bacterial virulence in macrophages^3^. Here, we identified another EsxG2/H2 pair, nearly identical to the EsxG/H, which is dependent on ESX-3 secretion, however it is encoded outside the *esx-3* locus. This suggests potential redundancy or similar biological functions for these heterodimers in *Mab*, warranting further investigation into their specific contributions to pathogenesis. Additionally, we observed that co-expression of the EspG3 chaperone is required for the secretion of PE5-PPE4 through ESX-3, supporting the formation of the PE5-PPE4-EspG3 heterotrimer structure, similar to the role of EspG5 in the secretion of the PE25/PPE41 heterodimer in *Mtb*^42,43^. PE/PPE proteins have been suggested to form channels in the mycomembrane, facilitating the selective uptake of small molecules^44,45^. Thus, it is possible that *Mab* PE5-PPE4 participates directly in the uptake of mycobactin siderophore-bound iron and the secretion of EsxG/H^18^. Supporting this hypothesis, recent findings indicate that Esx heterodimer secretion *via* ESX-5 depends on PE/PPE proteins forming channels in the mycomembrane^46^. Among the PE/PPE proteins translocated by the ESX-3, the MAB_0046/MAB_0047 pair is particularly notable for its high sequence identity to the PE5 and PPE4 from *Mtb*. Whether MAB_0046/MAB_0047 form channels and facilitate Esx substrate secretion remains to be demonstrated. ESX-5, present only in slow-growing mycobacteria, is responsible for the secretion of the majority of PE/PPE proteins in these species, including substrates not encoded within its locus^47,48^. However, some PE/PPE proteins are secreted by other ESX systems. For example, PPE68 is secreted by ESX-1^49^, while PE5 and PPE4 are secreted by ESX-3^3,39^. Unlike slow-growing mycobacteria, *Mab* lacks ESX-5 and its associated PE/PPE substrates. Instead, the only PE/PPE proteins present are PE5 and PPE4, which are secreted by ESX-3 in a manner consistent with its role in *Mtb*^3^. ESX-3 is conserved across mycobacterial species and is primarily associated with metal ion acquisition^4,39,50,51^. In *Mab*, ESX-3 does not replace the broad substrate secretion function of ESX-5 but instead fulfills a specialized role in secreting PE5 and PPE4, as well as highly homologous proteins as shown in this study.

**Fig. 7:**
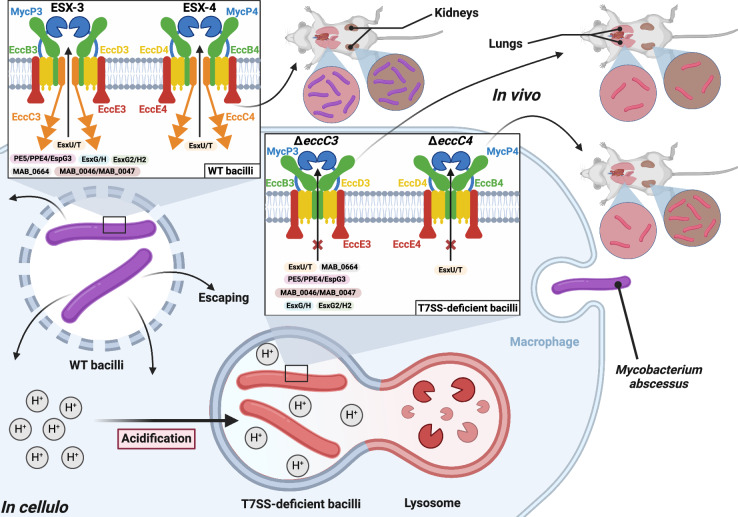
Effects of ESX-3 and ESX-4 depletion on *M. abscessus* pathogenesis. *Mab* possesses two T7SSs, ESX-3 and ESX-4. EccC3 and EccC4 are major membrane components of these machineries and are involved in substrate recognition. Following infection, WT bacilli are internalized in macrophage phagosomes and block fusion with acidic compartments. In these non-acidified compartments, ESX-secreted effectors, such as EsxU/EsxT, contribute to phagosomal membrane damage, allowing *Mab* to escape the phagosome and enter the cytosol. Here, the bacilli trigger the inflammasome, multiply, disseminate, and induce pathology, ultimately leading to the killing of the infected host. In this study, we show that both single and double *eccC* mutants most likely fail to block the phagolysosomal fusion process. In these mature phagosomes, ESX-3 and ESX-4 mutants cannot withstand the acidic pH and are eliminated by the macrophage, which can be linked to the incapacity to secrete effectors and/or to the inability to maintain metal homeostasis (for ESX-3). In mice, this translates into highly attenuated strains unable to colonize the lungs, thereby prolonging mice survival.

As expected, the secretion of EsxU/T was strongly reduced in the absence of EccC4. However, unlike the other substrates tested, EsxU/T secretion was also partially affected in the Δ*eccC3* strain. This indicates that, in the absence of ESX-4, the ESX-3 secretion machinery can partially compensate and secrete EsxU/T (Fig. 7). The defect in the secretion of EsxU/T in the Δ*eccC4* strain is consistent with previous observations of reduced protein levels in the culture supernatant of the Δ*eccB4* mutant^19^. This suggests that EsxU/T possesses structural features recognized by both ESX-3 and ESX-4. Since the C-terminus of EccC is implicated in substrate binding and recognition^52,53^, future work comparing the structures of different substrates^54,55^ may uncover common residues or structural elements involved in recognition by ESX-3 and ESX-4. While our homology-based approach identified new ESX substrates in *Mab* and confirmed their ESX-dependent secretion, this search was not exhaustive and additional substrates may remain undiscovered. Genome-wide analysis, combined with proteomic studies coupled with secretion assays in T7SS mutants, should help in elucidating the broad repertoire of ESX substrates in *Mab*.

Our findings indicate that both ESX-3 and ESX-4 are localized to the subpolar regions in *Mab*. Unlike most bacteria, mycobacteria exhibit asymmetric cell growth and division, elongating mainly from the polar regions where the new peptidoglycan is deposited^56,57^. The presence of ESX-3 and ESX-4 in this region of *Mab* may suggest an active assembly and secretion of substrates at the site of cellular expansion, further driving intracellular growth and enhancing pathogenesis upon infection.

Unlike most rapid-growing mycobacteria, *Mab* replicates inside macrophages by inhibiting phagosomal acidification^34^. Growth of Δ*eccC3* and Δ*eccC4* was attenuated in THP-1 cells, with Δ*eccC3* showing a more pronounced defect, particularly early in the infection process, likely due to a defect in internalization. Later in infection, both mutants showed decreased bacterial loads and fewer infected cells, which may, at least partly, be explained by increased co-localization with LysoTracker, suggesting that both ESX-3 and ESX-4 are needed to prevent fusion with acidic compartments and avoid phagosome maturation (Fig. 7). Previous studies using electron microscopy proposed that *Mab* causes phagosome membrane damage in macrophages, potentially facilitating its escape into the cytosol, aiding in bacterial multiplication and dissemination^34^. Here, the reduced co-localization of Δ*eccC3* and Δ*eccC4* mutants with galectin-3, a marker of phagosome membrane damage, supports the importance of both ESX systems in this process. Additionally, these mutants showed reduced secretion of IL-1β, consistent with a reduced phagosome-to-cytosol contact. Previous studies suggested that mycobacterial access to the cytosol triggers innate immune responses, including inflammasome activation and IL-1β release^58^. Aligning with findings from the Δ*eccB4* mutant^19^, our results suggest that ESX-3, like ESX-4, participates in damaging the phagosome membrane to establish phagosome-to-cytosol contact. The observation that the Δ*esxU/T* mutant of *Mab* could not induce phagosomal membrane damage, and the ability of purified EsxU/T complexes to interact and permeabilize artificial membranes^37^, suggest that the reduced EsxU/T secretion levels in Δ*eccC3* and Δ*eccC4* contribute to their impaired intracellular survival. However, this view is simplistic, given that ESX-3 secretes multiple effectors with unknown functions and that additional ESX-4-specific substrates remain to be identified. In *Mab*, the role of ESX-4 appears less pronounced than ESX-1 in *Mtb*^37,59^, likely due to factors such as the unique presence of the EccE4 protein^10^. Identifying additional ESX-4 substrates beyond EsxU/T will be essential to understand its contribution to virulence in *Mab*. The contrasting phenotypes of the *esxU/T* mutant (hypervirulence^37^) and *eccC4* mutant (hypovirulence) underscore the complexity of ESX-4, which likely influences virulence through both specific substrates and system-wide mechanisms.

The essential nature of *esx-3* in *Mtb* prevents the characterization of its unique role during pathogenesis, whereas its dispensability in *Mab* enables such studies. Previous, Kim et al. deleted the entire *esx-3* locus in *Mab*, resulting in attenuated virulence in mice^16^. However, this deletion also removed key genes such as *esxG/esxH* and *pe4-ppeE5-espG3* making it unclear whether the attenuation was due to the loss of the ESX-3 machinery and/or the secreted effectors. In contrast, the study did not include complementation experiments to validate the observations. Our study takes a different approach by specifically deleting the ATPase component of each ESX system, allowing for a targeted assessment of the virulence profile for each ESX mutant. The most prominent results from our mice experiments show that both ESX-3 and ESX-4 contribute equally to *Mab* virulence in the lungs. No significant changes in bacterial burden were noted in the spleen and liver compared to WT *Mab* infected animals. Conversely, Δ*eccC3*, but not Δ*eccC4*, was completely eradicated from the kidneys. Importantly, mice infected with WT *Mab* succumbed rapidly, whereas those infected with any of the deletion mutants survived, showing no signs of pathology and no or reduced body weight loss. This indicates that both ESX-3 and ESX-4 are required for lung colonization and pathogenesis of *Mab* in mice. Of note, the severe attenuation of the ESX-3 mutant in macrophages and mice is likely due to the loss of effector secretion but also to the impaired metal homeostasis^3^.

In conclusion, our study reports the first mycobacterial strain devoid of all T7SS. We demonstrate that the simultaneous loss of ESX-3 and ESX-4 in *Mab* is dispensable for in vitro growth but results in pronounced attenuated phenotypes in macrophages and mice, which were further exacerbated in the Δ*eccC3/*Δ*eccC4* mutant. Thus, we propose that Δ*eccC3* or Δ*eccC3/*Δ*eccC4* could serve as potential live attenuated vaccine candidates against *Mab* diseases. Future studies should focus on evaluating the safety and protective efficacy of these strains against a challenge with virulent *Mab* strains.

## Methods

### Mycobacterial strains, growth conditions, and reagents

All mycobacterial strains used in this study are listed in Supplementary Table 2. *Mab* CIP104536^T^, R and S variants were typically grown in Middlebrook 7H9 broth (BD Difco) supplemented with 0.05% Tween 80 and 10% OADC enrichment (oleic acid, albumin, dextrose, catalase; BD Difco) (7H9^OADC^). Cultures were maintained at 37 °C, with antibiotics added as necessary. For transformation, electrocompetent mycobacteria were subjected to electroporation using a Bio-Rad Gene Pulser with settings of 25 µF, 2500 V, and 800 Ohms. Bacterial selection was achieved by supplementing the media with 1 mg/mL hygromycin for strains carrying pTEC27^60^ (Addgene, plasmid 30182), which allows for tdTomato expression, or with 250 µg/mL kanamycin for strains harboring the pMV306 derivatives. For colony selection on solid media, Middlebrook 7H10 agar (BD Difco) supplemented with 10% OADC enrichment (7H10^OADC^), LB agar, or Tryptic Soy Broth agar (Sigma) were used. Antibiotics were obtained from Sigma-Aldrich.

### Bacterial growth

For growth experiments, pre-cultures in the exponential phase were diluted to an optical density (OD) at 600 nm of 0.001 in glycerol-alanine-salts medium, without iron citrate, and supplemented with 0.05% Tween 80^18^ or in 7H9^OADC^. A volume of 200 µL of this culture was transferred to a treated flat-bottom 96-well plate (Corning, New York, USA). The plates were incubated statically at 37 °C for 7 days. Daily measurements of bacterial growth were taken using a spectrophotometer multimode microplate reader (Tecan Spark 10 M; Tecan Group Ltd., Switzerland). Each experiment included six technical replicates and three biological replicates per strain, resulting in a total of 18 samples per condition.

### Deletion of *eccC* genes

To generate unmarked single and double deletion mutants in *Mab* smooth (S) and rough (R) morphotypes, we used the suicide vector pUX1-*katG*^22,61^. The deletion process involved amplifying the left and right arms (LA and RA) flanking the *eccC3* and *eccC4* genes using genomic DNA, Q5 polymerase (New England Biolabs), and specific primers (Supplementary Table 1). For *eccC3* gene deletion, the LA was amplified with primers 1/2, and the RA with primers 3/4. The resulting PCR products were purified, digested with PacI/MfeI for the LA and MfeI/NheI for the RA, and then ligated into the PacI/NheI-linearized pUX1-*katG*, yielding pUX1-*katG-eccC3*. This construct was designed to delete 3780 bp (94%) of the *eccC3* open reading frame. Similarly, pUX1-*katG-eccC4* was constructed to remove 3681 bp (93%) of the *eccC4* ORF. Electrocompetent *Mab* were transformed with these constructs to generate Δ*eccC3* and Δ*eccC4*. For the double mutant, Δ*eccC3* was used as a base to remove *eccC4*. Bacteria that underwent the first homologous recombination event were identified based on red fluorescence on 7H10^OADC^ agar supplemented with 250 µg/mL kanamycin. Following an overnight subculture in 7H9^OADC^ without kanamycin, cultures on 7H10^OADC^ containing 50 µg/mL INH were plated and non-fluorescent colonies screened for INH-resistance and kanamycin-sensitivity. Genotypes of the mutants were confirmed by sequencing the DNA junctions with primers listed in Supplementary Table 1.

### Complementation of *eccC* mutants

To complement the *eccC* mutants, plasmids were constructed by PCR amplifying the *eccC3* and *eccC4* genes fused either to HA or to mNeonGreen tags under the control of the *hsp60* promoter. Amplicons were ligated into the integrative vectors pMV306, pMV306-*eccC3-mNeonGreen*, pMV306-*eccC4-mNeonGreen*, which were linearized with the appropriate restriction enzymes (Supplementary Table 1, and Supplementary Table 3). Alternatively, the In-Fusion cloning method was used. The resulting vectors, pMV306-*eccC3* and pMV306-*eccC4*, were sequenced to verify their correctness and then introduced into the respective Δ*eccC3* and Δ*eccC4* mutants. The complementation constructs are listed in Supplementary Table 3.

### Constructs containing ESX system substrates

These vectors were generated using the primers listed in Supplementary Table 1. pMV306 was used to create vectors capable of expressing the various ESX substrates, either individually, in pairs or in triplets, fused with either an HA or a Strep tag placed at the C-terminus. Gene expression was controlled under the *hsp60* promoter. In total, six distinct constructs were generated, featuring the following gene combinations: *esxG*/*esxH*, *esxG2*/*esxH2*, *pe5*/*ppe4*/*espG3*, *esxU*/*esxT*, *MAB_0664*, and *MAB_0665*/*MAB_0666* using MfeI and HpaI for pMV306-*esxG/esxH-HA*, pMV306-*esxG2/esxH2-HA*, pMV306-*ppe4-HA*, pMV306-*MAB_0664-HA*, and pMV306-*MAB_0665/MAB_0666-HA* and MfeI and HindIII for pMV306-*esxU/esxT-HA*. Plasmid pMV306-*pe5/ppe4-HA/espG3-Strep* was generated using the In-Fusion method (TAKARA). This involved to sequentially inserting the *pe5* gene upstream of *ppe4*, followed by the *espG3* gene downstream of *ppe4*. All constructs were sequenced and introduced into various *Mab* strains.

### Localization of EccC3 and EccC4

To study the localization of EccC3 and EccC4, *eccC3* and *eccC4* were fused with the gene encoding the mNeonGreen fluorescent protein and constructs introduced into Δ*eccC3* and Δ*eccC4* strains. Transformants were routinely grown at 37 °C in Middlebrook 7H9 supplemented with 0.2% glycerol, 10% OADC, 0.05% Tween-80, and 250 µg/mL kanamycin. Cultures were continuously shaken to ensure proper aeration. For imaging, cells were grown to an OD_600_ of 0.6-0.8 and imaged live using super-resolution fluorescence light microscopy. The imaging was conducted with a Plan-Apochromat 100×/1.46 oil objective lens on a Zeiss LSM 900 confocal microscope equipped with an AiryScan 2 detector and a Colibri 5 light source. This system allows precise localization of fluorescently-tagged EccC proteins by providing 160 nm lateral resolution and ~400 nm axial resolution with 8× improved signal-to-noise ratio compared to regular confocal microscopy.

### Subcellular fractionation

Mycobacterial cultures (500 mL) of Δ*eccC3*::*eccC3*-mNeonGreen, Δ*eccC4*::*eccC4*-mNeonGreen, Δ*eccC3*::*eccC3*-HA, and Δ*eccC4*::*eccC4*-HA were grown in 7H9 medium supplemented with 1% glucose at 37 °C with shaking until reaching OD_600_ of ~0.8. Cells were harvested by centrifugation at 10,000 × *g* for 30 min, washed with PBS containing 1 mM benzamidine, and resuspended in 2 mL of the same buffer. Bacterial cells were lysed using a bead beater, and the lysates were transferred to 15 mL tubes for two rounds of sonication (30 s each on ice). The lysates were then centrifuged twice at 4000 × *g* for 10 min to remove intact cells. A 200 μL aliquot of each resulting supernatant was stored at 4 °C as a total lysate sample. The remaining supernatants were subjected to centrifugation at 16,000 × *g* for 30 min at 4 °C to pellet cell walls (peptidoglycan, arabinogalactan, and outer membrane layers). The resulting supernatants were further ultracentrifuged at 200,000 × *g* for 2 hrs at 4 °C to separate plasma membranes (pellet) from the cytoplasmic fraction. The cell wall and plasma membrane fractions were each washed twice with PBS supplemented with 1 mM benzamidine and then resuspended in 200 μL of PBS containing protease inhibitors. The cytoplasmic fractions underwent an additional ultracentrifugation step at 200,000 × *g* for 2 hrs to ensure removal of residual membranes. Protein concentrations in each fraction were determined using a BCA kit, and 20 μg of protein from each fraction was loaded onto SDS-PAGE gels for Western blotting.

### Western blotting

Mycobacterial cultures were grown in 7H9 broth. Bacteria were harvested by centrifugation at 3000 × *g* for 10 min at 4 °C, washed, and resuspended in cold PBS supplemented with a protease inhibitor cocktail (Sigma-Aldrich). Bacteria were lysed by adding 1-mm-diameter glass beads and subjected to two cycles of 3-min pulses at full speed using a bead-beater device (Mixer Mill MM 301, Retsch). Lysates were collected, and protein concentrations were determined using the BCA Protein Assay Reagent kit (ThermoFisher Scientific), following the manufacturer’s instructions. Equal amounts of proteins were separated by 10% SDS-PAGE and transferred onto a polyvinylidene fluoride membrane (Immobilon-P, Merck Millipore). For protein detection, membranes were probed with rat anti-HA antibodies (1:3000 dilution) or mouse monoclonal antibodies 32/15 against the Ag85 complex (1:40 dilution) or mouse anti-GroEL2 antibodies (1:5000 dilution) or rat anti-KasA antibodies (1:2000) or rabbit anti-mNeonGreen antibodies (1:2000, Cell Signaling). After primary antibody incubation, membranes were washed and incubated with anti-rat or anti-mouse or anti-rabbit secondary antibodies conjugated to horseradish peroxidase (HRP) (1:5000 or 1:20000). Protein bands were visualized using the HRP reaction with SuperSignal West Femto kit (ThermoFisher Scientific) and imaged on a ChemiDoc MP system (Bio-Rad).

### Secretion assays

Cultures were cultivated in 7H9^OADC^ until saturation. Bacteria were washed and transferred into Sauton’s medium, grown to an OD_600_ of 2, and then pelleted at 3000 g for 20 min. The pellets were washed and resuspended in phosphate-buffered saline (PBS) before being lysed by bead beating. A ¼ volume of 4x Laemmli buffer was added, yielding the pellet fraction. The resulting supernatants were collected, passed through a 0.2-µm filter, supplemented with 1 mM PMSF, and concentrated approximately 200-fold using an Amicon 3-kDa centrifugal filter (Merck Millipore). The total protein concentration was determined using the Pierce BCA protein assay kit (ThermoFisher Scientific). Proteins from both the pellet fraction and culture filtrates were separated by SDS/PAGE, and the presence of ESX-3 and/or ESX-4-secreted substrates was assessed by Western blotting using rat anti-HA antibodies (dilution 1/3000) (Roche). After washing, the membranes were incubated with goat anti-rat antibodies conjugated to horseradish peroxidase (HRP) at a 1:5000 dilution (Abcam). Protein bands were developed using the SuperSignal West Femto kit (Thermo Scientific) and visualized with the ChemiDoc MP imaging system (Bio-Rad Laboratories).

### Quantification of secreted proteins

Bands corresponding to secreted proteins identified by Western blotting were quantified using ImageJ. First, the image file was opened in ImageJ (*File > Open*). To ensure compatibility with the analysis, the image was converted to grayscale if necessary. Using the rectangular selections tool from the ImageJ toolbar, a narrow rectangle was drawn around the lanes, ensuring that they encompassed only a single vertical lane (with individual bands running horizontally). Then the *Analyze > Gels > Select First Lane* was used to set the rectangle in place, marking the lanes with a highlighted region and a number “1” in its center. Subsequently, *Analyze > Gels > Plot Lanes* was selected to generate a profile plot of the signal intensity across the lanes. To quantify individual bands, the *Straight-Line Selection* tool was used to close off the base of each peak in the profile plot. Then, the *Wand* tool from the ImageJ toolbar was used to click inside each peak, sequentially highlighting them. As each peak was selected, the integrated density values were displayed in the results window. The raw data from the results window were transferred to a spreadsheet for further analysis. The relative density of each band was calculated by normalizing to the corresponding loading control or reference band. The processed data were subsequently visualized and plotted using GraphPad Prism.

### Colony-biofilm

The colony-biofilm model was carried out as reported previously^25^. Cultures were normalized to an OD_600_ of 0.2, and 10 μL of each culture was pipetted onto sterile nitrocellulose filter membranes (13 mm, 0.2 μm pore size). These membranes were then deposited on Middlebrook 7H11 agar supplemented with 2.5% BSA, 0.85% NaCl and 2% dextrose. Plates were incubated at 37 °C under humidified conditions for 5 days. To quantify the number of CFU/cm^2^ on the sterile membranes after 4 days, the colony-biofilms were transferred to 10 mL of PBS  +  0.025% tyloxapol and vortexed with glass beads to disperse the cells. After vortexing, the samples were sonicated for 5 min at room temperature. For R strains, the sonication was repeated twice. The resulting suspensions were serially diluted and plated on LB agar to enumerate CFUs.

### H_2_O_2_ disk diffusion method

This method was used to qualitatively measure the differences in H_2_O_2_ susceptibility between the strains. Cultures grown for 72 hrs in Middlebrook 7H9 supplemented with 10% OADC and 0.025% tyloxapol were diluted to an OD_600_ of 0.5 in PBS containing 0.025% tyloxapol. The diluted cultures were then plated to form a bacterial monolayer on Mueller-Hinton agar. To evaluate H_2_O_2_ susceptibility, 20 µL of 30% H_2_O_2_ was spotted onto 6-mm-diameter Whatman disks, which were subsequently placed on the bacterial lawn. After 72 hrs of incubation at 37 °C, the inhibition zones (halos) were measured.

### Macrophage infection

THP-1 cells were cultured in RPMI medium supplemented with 10% fetal bovine serum (FBS; Sigma-Aldrich) and incubated at 37 °C in a 5% CO_2_ atmosphere. To differentiate these cells into macrophages, 20 ng/mL of phorbol myristate acetate was added to the cultures in 24-well flat-bottom tissue culture microplates, at a density of 10^5^ cells/well, and incubated for 48 hrs under the same conditions. For the infection assay, wild-type and mutant strains carrying pMV306-mScarlet^62^ and the complemented strain carrying pTEC27^60^, were used to infect macrophages at a MOI of 2:1. The infection was conducted for 4 hrs at 37 °C with 5% CO_2_. After infection, cells were washed with PBS and incubated in RPMI/FBS containing 250 µg/mL of amikacin for 2 hrs to eliminate extracellular bacteria. The amikacin-containing medium was then aspirated, and the cells washed three times with PBS. To assess the bacterial burden, the infected macrophages were incubated with RPMI medium containing 50 µg/mL amikacin at 37 °C. At 4, 24, and 72 hpi, macrophages were washed and lysed with 100 µL of 1% Triton X-100. Lysis was stopped by adding 900 µL PBS, and serial dilutions of the lysates were plated on LB agar to determine intracellular bacterial counts. CFUs were counted after 5 days of incubation at 37 °C.

### Adhesion assay

To assess bacterial adhesion, 100,000 macrophages were seeded into each well of a 24-well plate and placed on ice for 30 min. Bacteria were then added at a MOI of 100:1. After a 30 min incubation on ice, the cells were washed 3 times with 1 mL of cold PBS to remove non-adherent bacilli. Subsequently, 1 mL of cold water was added to the wells, and the plates were refrigerated for 45 min. Cells were lysed by adding 0.1 mL of 0.1% Triton X-100. The lysates were diluted in PBS and plated on LB agar for CFU counting.

### Immunofluorescence staining of infected macrophages

For microscopy-based infection assays, THP-1 cells were cultivated on coverslips in 24-well plates at a density of 10^5^ cells/well and incubated for 48 hrs at 37 °C with 5% CO_2_. Cells were then infected with either mScarlet- or tdTomato-expressing *Mab* (MOI 2:1) for 4 hrs, washed, treated with amikacin, and fixed 24 hpi with 4% paraformaldehyde in PBS for 20 min. Following fixation, the cells were permeabilized using 0.2% Triton X-100 for 20 min, blocked for 20 min with 2% BSA in PBS supplemented with 0.2% Triton X-100, and then incubated with an anti-CD43 antibody (Becton Dickinson; dilution 1:1000) for 1 hr, followed by incubation with an Alexa Fluor 488-conjugated anti-mouse secondary antibody (Molecular Probes, Invitrogen). After washing with PBS, the cells were mounted onto microscope slides using Immumount (Calbiochem) and examined using a confocal microscope equipped with a 40x objective (Zeiss LSM880). The LSM880 confocal microscope is equipped with an Airyscan detector and advanced laser light sources. This system allows precise protein localization, providing ~140 nm lateral resolution and ~400 nm axial resolution, with up to a 4x improvement in signal-to-noise ratio compared to standard confocal microscopy.

### Phagosomal acidification assay

THP-1 cells were seeded onto glass coverslips in 24-well plates and infected with fluorescent *Mab* strains for 3 hrs at 37 °C with 5% CO_2_. Following infection, cells were treated with amikacin (250 μg/mL) to eliminate extracellular bacteria. Green LysoTracker (1/2000 dilution, Thermo Fisher Scientific) was subsequently added to each well during 17 hrs. The colocalization of red fluorescent *Mab* within cells and LysoTracker green was assessed by analyzing 900 infected cells under a confocal microscope.

### Galectin-3 immunostaining

THP-1 cells were seeded on coverslips in 24-well plates at a density of 10^5^ cells/well. Cells were infected with fluorescent *Mab* strains (MOI 10:1) for 3 hrs, washed, and treated with amikacin. At 20 hpi, cells were fixed with 4% paraformaldehyde in PBS for 15 min. Phagosomal membrane damage was assessed by immunostaining using a purified mouse anti-human Galectin-3-specific monoclonal antibody (555746; BD Pharmingen) at a 1:1000 dilution, followed by incubation with a 488-conjugated anti-mouse secondary antibody (Invitrogen). Images were acquired using a Zeiss Axioimager confocal microscope equipped with a 40x or 63x oil objective and processed with Zeiss Axiovision software. An infected cell was considered positive if at least one mycobacteria-containing phagosome was stained positive for galectin-3 alongside *Mab*. Galectin-3-positive signals predominantly localized to membranous structures surrounding bacteria-containing phagosomes. The percentage of infected cells with galectin-3-positive *Mab*-containing phagosomes was quantified from at least 900 infected cells.

### Immunofluorescence staining of phagosomal damage

THP-1 cells were cultured on coverslips, infected, and fixed according to standard procedures. Phagosomal membrane damage was detected in green using antibodies against ubiquitinated proteins (FK2, Sigma), diluted in blocking buffer. The antibodies were visualized with secondary antibodies conjugated to Alexa Fluor 488 (Invitrogen). *Mab* was identified by its red autofluorescence, as indicated above. An infected cell was considered positive if at least one of its *Mab*-containing phagosomes stained positive for the phagosomal damage marker. The percentage of infected cells with at least one *Mab*-containing phagosome positive for ubiquitinated protein was determined from 900 cells.

### IL-1β ELISA

Supernatants from THP-1/*Mab* co-cultures (MOI 10:1) were harvested at 20 hpi and analyzed for human IL-1β secretion using the IL-1β ELISA kit (Biolegend), following the manufacturer’s protocol.

### Ethics statement

All procedures involving mice were conducted in strict adherence to ethical guidelines and were approved by the Animal Experimentation Ethics Committee (N°047, Comité d’éthique Pelvipharm, Montigny-le-Bretonneux, France, agreement A783223) as well as the French Ministry of Higher Education, Research and Innovation (MESRI) under APAFIS #44601-2023090514417206 v3. Three-month-old male and female *Mus musculus* C3HeB/FeJ mice were used in this study and were housed with unrestricted access to food and water, in compliance with European animal welfare regulations. We have complied with all relevant ethical regulations for animal use. Additionally, adherence to these ethical standards was monitored throughout the experiments by the animal welfare structure of the animal facilities platform.

### Mice infection and bacterial load determination

To evaluate the impact of infection on mortality and bacterial burden, *C3HeB/FeJ* mice (initial weight ~36 g) were intravenously infected *via* the tail vein with 2 × 10^7^ CFU per mouse in 200 µL of 0.9% NaCl solution. The mice were infected with the parental *S* strain, single mutants (*SΔeccC3* and *SΔeccC4*), their respective complemented strains, or the double mutant (*SΔeccC3/ΔeccC4*). At 1, 7, 14, and 21 days post-infection, mice were euthanized, and their organs were harvested, homogenized, serially diluted, and plated onto VCAT (vancomycin, colistin sulfate, amphotericin B, and trimethoprim) chocolate agar plates (bioMérieux, France). The plates were incubated at 37 °C for 5–6 days before CFU enumeration. Bacterial loads were expressed as log10 CFU ± SD. A total of five mice per group (three for day 1) were included in the study.

### Statistics and reproducibility

Data were analyzed using Zen software (black edition, ZEISS), Image J-Fiji software (NIH), or GraphPad Prism 10.2.2 (GraphPad, La Jolla, CA, USA), with details provided in each figure legend. Statistics in text or figures represent mean ± SD. The significance of observations across multiple groups was assessed using one-way ANOVA or two-way ANOVA, followed by Mann Whitney, Mental-Cox, or Tukey’s post-hoc test. Results are denoted as: ns, non-significant; *, *P* < 0.05; **, *P* < 0.01; ***, *P* < 0.001; ****, *P* < 0.0001. Each experiment was repeated at least 2 times independently.

### Reporting summary

Further information on research design is available in the Nature Portfolio Reporting Summary linked to this article.

## Supplementary information

Supplementary Material

Supplementary Data 1

Description of Additional Supplementary File

Reporting summary

## Data Availability

The source data behind the graphs in the paper can be found in Supplementary Data 1. Uncropped western blots are included in the Supplementary information. All other data supporting the findings of this study are available from the corresponding author upon request.
