# Supplementary material for: Deletion of ESX-3 and ESX-4 secretion systems in Mycobacterium abscessus results in highly impaired pathogenicity
Source: Commun Biol. 2025 Feb 3;8:166. doi: 10.1038/s42003-025-07572-4 (PMC11791044; doi:10.1038/s42003-025-07572-4)
Supplement: Supplementary file 1 — Supplementary Material [file 42003_2025_7572_MOESM1_ESM.pdf]

Supplementary Information

Deletion of ESX-3 and ESX-4 secretion systems in *Mycobacterium abscessus* results in highly impaired pathogenicity

Wassim Daher[1,2*], Vincent Le Moigne[3$], Yara Tasrini[1$], Shweta Parmar[4], Danielle L. Sexton[4], John Jairo Aguilera-Correa[1], Valentin Berdal[1], Elitza I. Tocheva[4], Jean-Louis Herrmann[3], and Laurent Kremer[1,2*]

[1]Centre National de la Recherche Scientifique UMR 9004, Institut de Recherche en Infectiologie de Montpellier (IRIM), Université de Montpellier, 1919 route de Mende, 34293, Montpellier, France.

[2]INSERM, IRIM, 34293 Montpellier, France.

[3]Université Paris-Saclay, UVSQ, Inserm, Infection et inflammation, 78180, Montigny-Le-Bretonneux, France.

[4]Department of Microbiology and Immunology, University of British Columbia, Vancouver, Canada.

**Corresponding author:* *Wassim Daher, E-mail:* wassim.daher@irim.cnrs.fr; *Laurent Kremer, E-mail:* laurent.kremer@irim.cnrs.fr

8 Supplementary Figures
3 Supplementary Tables
Uncropped Western blots from Main Figures and Supplementary Figures

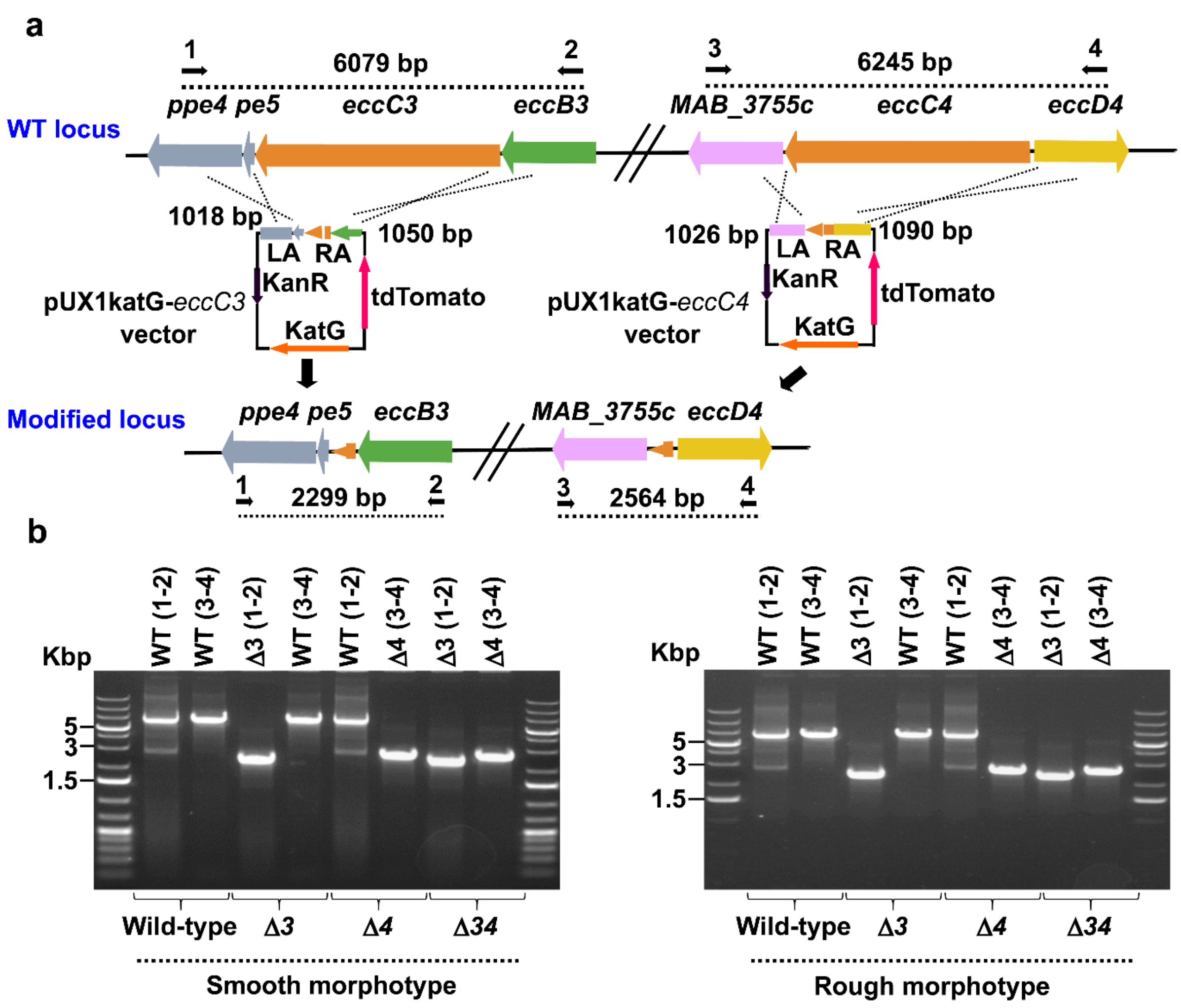

Supplementary Figure 1. Deletion of *eccC3* and/or *eccC4* in *M. abscessus.* (a) The *eccC3* gene is sandwiched between *eccB3* and *pe5/ppe4*, while the *eccC4* gene is wedged between *eccD4* and *MAB_3755c.* Plasmids pUX1-*katG-eccC3* and pUX1-*katG-eccC4* were generated to remove the *eccC3* and *eccC4* genes by double homologous recombination. Genomic fragments of 1018 bp and 1026 bp corresponding to the left arm (LA) DNA sequences of *eccC3* and *eccC4*, respectively, were amplified by PCR and sub-cloned into the PacI and MfeI sites of pUX1-*katG*. The right arm (RA) DNA sequences of *eccC3* and *eccC4* were amplified from genomic DNA by PCR (1050 bp and 1090 bp, respectively) and cloned into the MfeI and NheI sites of pUX1-*katG*. The resulting suicide plasmids, which lack motifs for episomal replication or mobile elements promoting chromosomal integration, were used to transform *Mab* S and R. These plasmids could only propagate *via* homologous recombination between the cloned sequences and their chromosomal homologous sequences. The first recombination events were selected in the presence of kanamycin. Single red fluorescent tdTomato-expressing clones were subjected to a second round of recombination, selected on isoniazid, and screened for double

crossover phenotypes, indicated by the loss of red fluorescence, sensitivity to kanamycin, and resistance to isoniazid. Dotted lines represent the size (indicated above each line) of the expected PCR products in the parental *Mab* WT strain and Δ*eccC3* and Δ*eccC4* mutants. Black arrows represent the primers used for PCR analysis. (**b**) PCR analysis demonstrating the deletion of Δ*eccC3*, Δ*eccC4*, and Δ*eccC3/*Δ*eccC4* in the various strains. Genomic DNA from WT bacteria was used to amplify the intact loci. Amplicons were sequenced to confirm the proper deletion of the genes.

a b

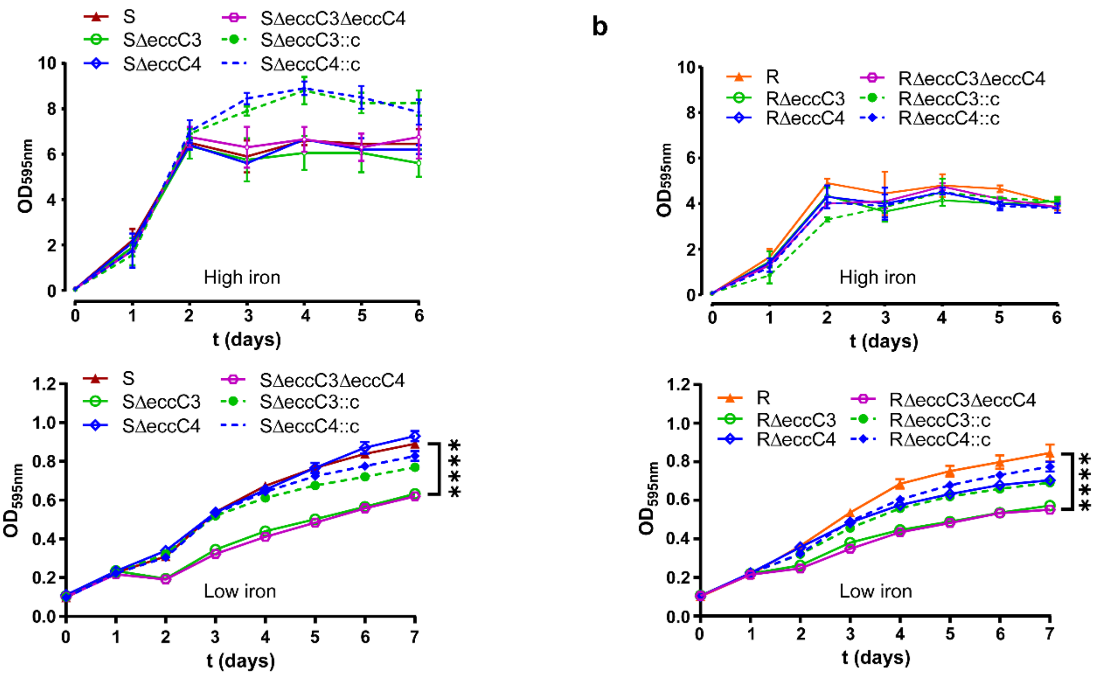

c

Strain	Slope reduction respect S (%)	p-value
S	0	1.000
SΔeccC3	37,2	<0,0001
SΔeccC4	-2,07	<0,0001
SΔeccC3 ΔeccC4	37,8	<0,0001
SΔeccC3::c	17,71	<0,0001
SΔeccC4::c	8,88	<0,0001
**Strain**	**Slope reduction respect R (%)**	**p-value**
R	0	1.000
RΔeccC3	40,8	<0,0001
RΔeccC4	21,65	<0,0001
RΔeccC3 ΔeccC4	41,54	<0,0001
RΔeccC3::c	22,98	<0,0001
RΔeccC4::c	10,77	<0,0001

d e

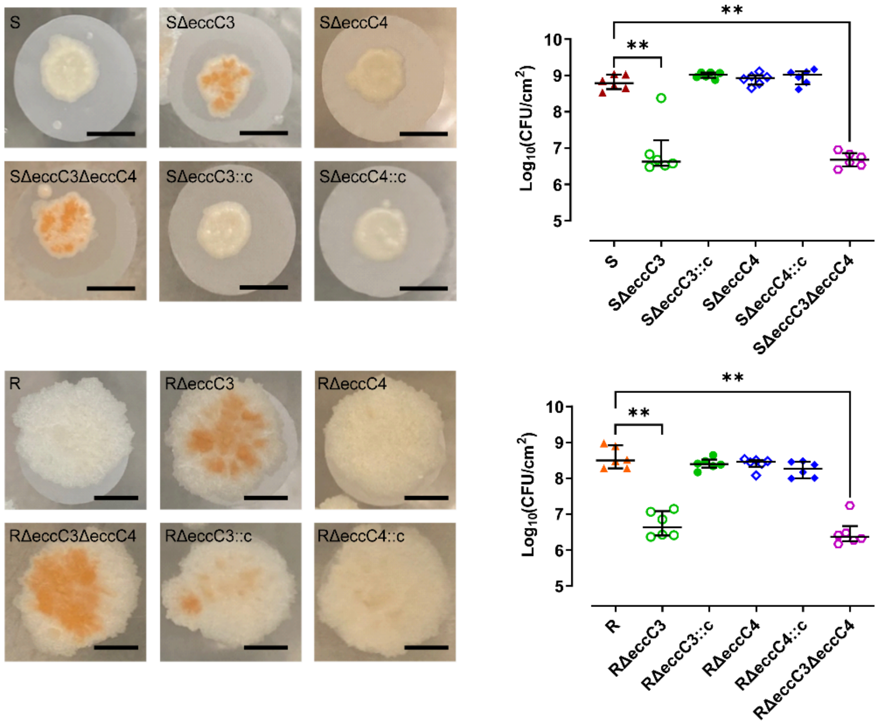

49

Supplementary Figure 2. *In vitro* growth of *M. abscessus esx* mutants and complemented strains in planktonic cultures and biofilms. (**a**) Growth of S-derived mutants and complemented strains in Middlebrook 7H9 medium (upper panel) or under iron-limited conditions in GAST medium (lower panel). (**b**) Growth of R-derived mutants and complemented strains. The bars represent the standard deviation. Growth was monitored by measuring OD_{595} nm for 7 days. For (**a**) and (**b**), the experiments were conducted with two biological replicates, each having six technical replicates (n=12). *P*-values were calculated using ANOVA with Tukey's post-hoc test; ****, $P<0.0001$. (**c**) Pairwise comparisons of linear regression slopes for each strain relative to its parental strain. (**d**) Colony-biofilm appearance of *Mab* S-derived strains. Biofilm-supporting membranes were incubated at 37 °C under humidified conditions for 5 days (left panel). Scale bar, 5 mm. Quantification of CFU/cm^2 was performed after 4 days (right panel). (**e**) Colony-biofilm appearance of *Mab* R-derived strains. Biofilm-supporting membranes were incubated at 37 °C under humidified conditions for 5 days (left panel). Quantification of CFU/cm^2 was performed after 4 days (right panel). Each experiment was performed in triplicate with two technical replicates and three biological replicates per strain (n=6). Scale bar, 5 mm. For (**d**) and (**e**), the error bars represent the interquartile range. **, $P<0.01$. Data are mean ± SD.

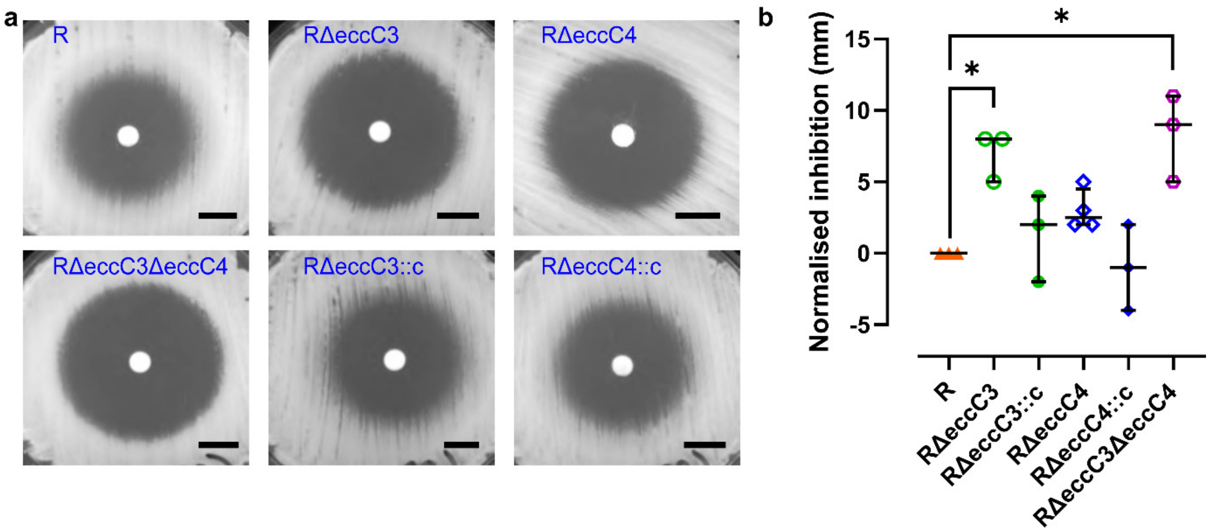

Supplementary Figure 3. H_2O_2 susceptibility of R-derived ESX strains. H_2O_2 was spotted on 6-mm-diameter Whatman disks and placed on the bacterial lawn. After 72 hrs of incubation at 37 °C, pictures were taken (scale bar, 1 cm) (**a**), and the diameter of the halo was measured (**b**). All experiments were performed in triplicate (n=3). Results are expressed as the median and interquartile range of the diameter (mm) of each strain subtracted from the wild-type R strain. *P*-values were calculated using ANOVA with Tukey's post-hoc test; *, $P<0.05$. Data are mean ± SD.

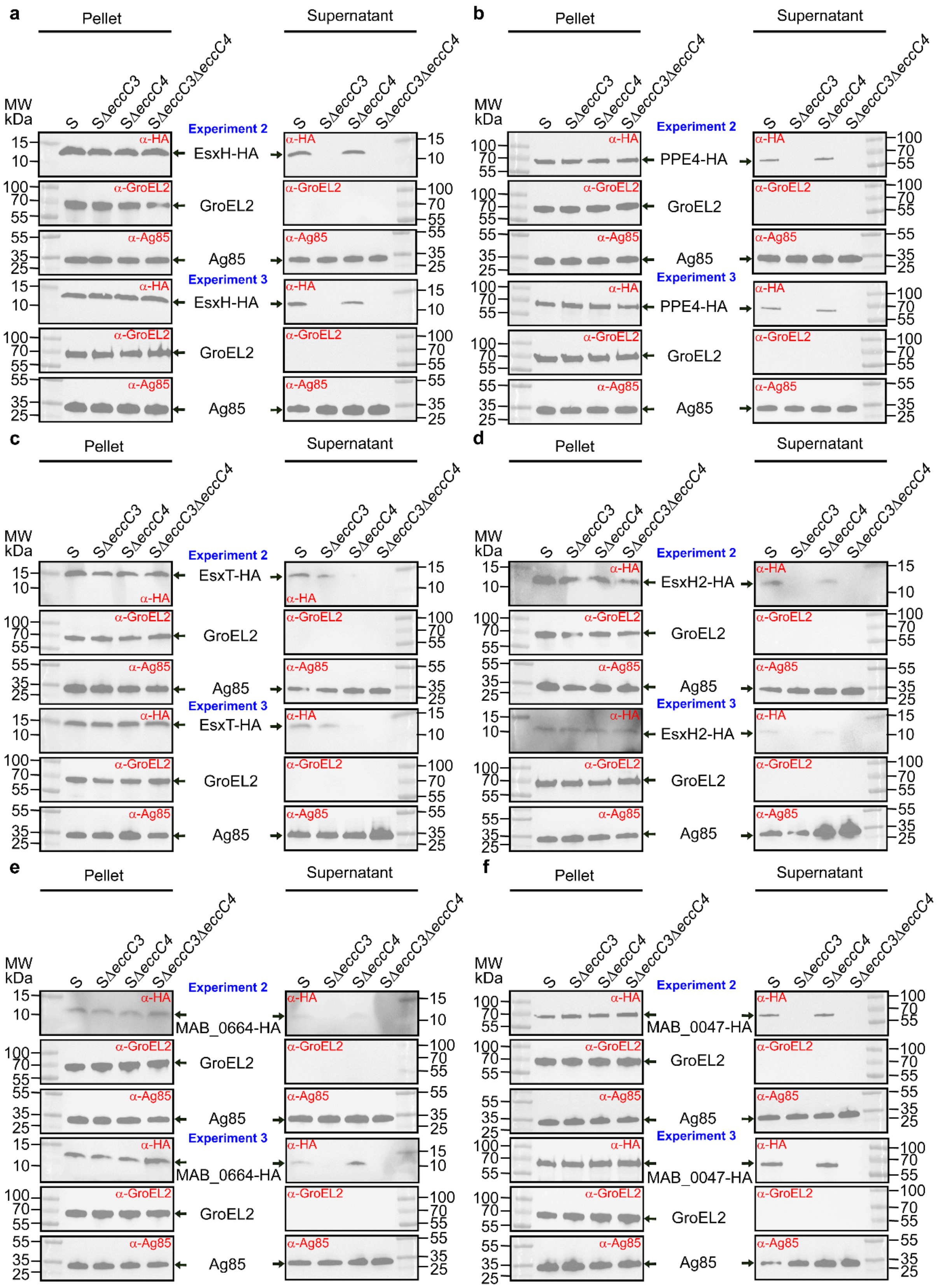

Supplementary Figure 4. Differential roles of ESX-3 and ESX-4 in substrate secretion. (a-f) Western blot analysis of bacterial total lysates and corresponding culture supernatants highlighting the distinct roles of ESX-3 and ESX-4 in substrate secretion. Ag85 is used as a

loading control for total lysates and to confirm that its secretion remains unaffected in the ESX mutants. GroEL2 serves as a loading control for total lysates and as a marker to verify the absence of bacterial lysis, ensuring that protein detection in the culture supernatants is not artifactual. This figure presents data from experimental replicates 2 and 3.

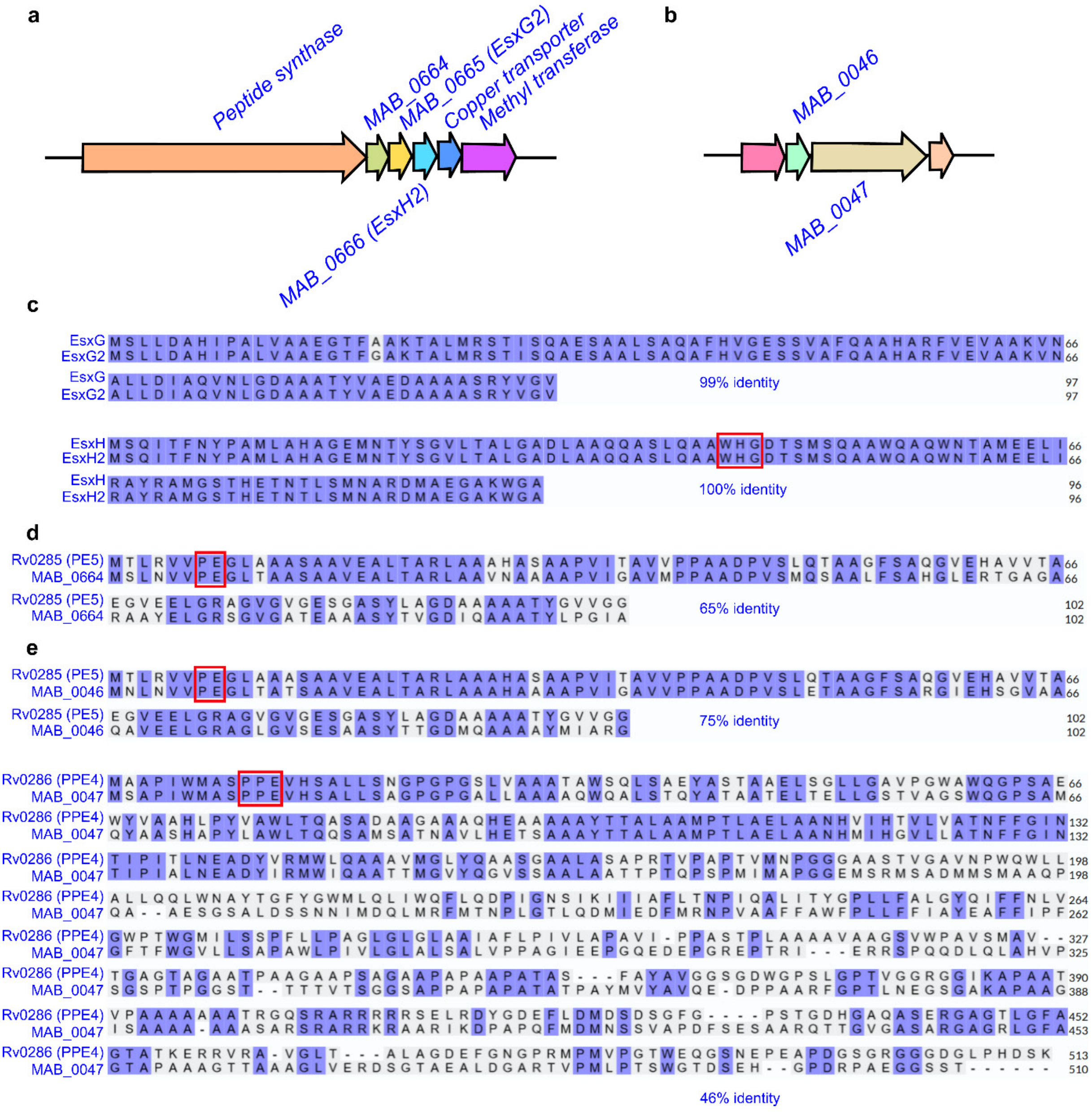

Supplementary Figure 5. Identification of new ESX substrates in *M. abscessus*. (**a**) *Mab* genomic locus comprising *MAB_0664, MAB_0665*, and *MAB_0666* located outside the *esx* loci. (**b**) *Mab* genomic locus comprising *MAB_0046* and *MAB_0047* located outside the *esx* loci. (**c**) Amino-acid alignments between EsxG and MAB_0665 (EsxG2), and EsxH and MAB_0666 (EsxH2). (**d**) Amino-acid alignments between Rv0285 (PE5) and MAB_0664. (**e**) Amino-acid alignments between Rv0285 (PE5) and MAB_0046, and Rv0286 (PPE4) and MAB_0047. The PE and PPE signatures, as well as the WxG motif, are shown in a red box. Sequences were retrieved from Mycobrowser and aligned using Clustal Omega and Jalview software.

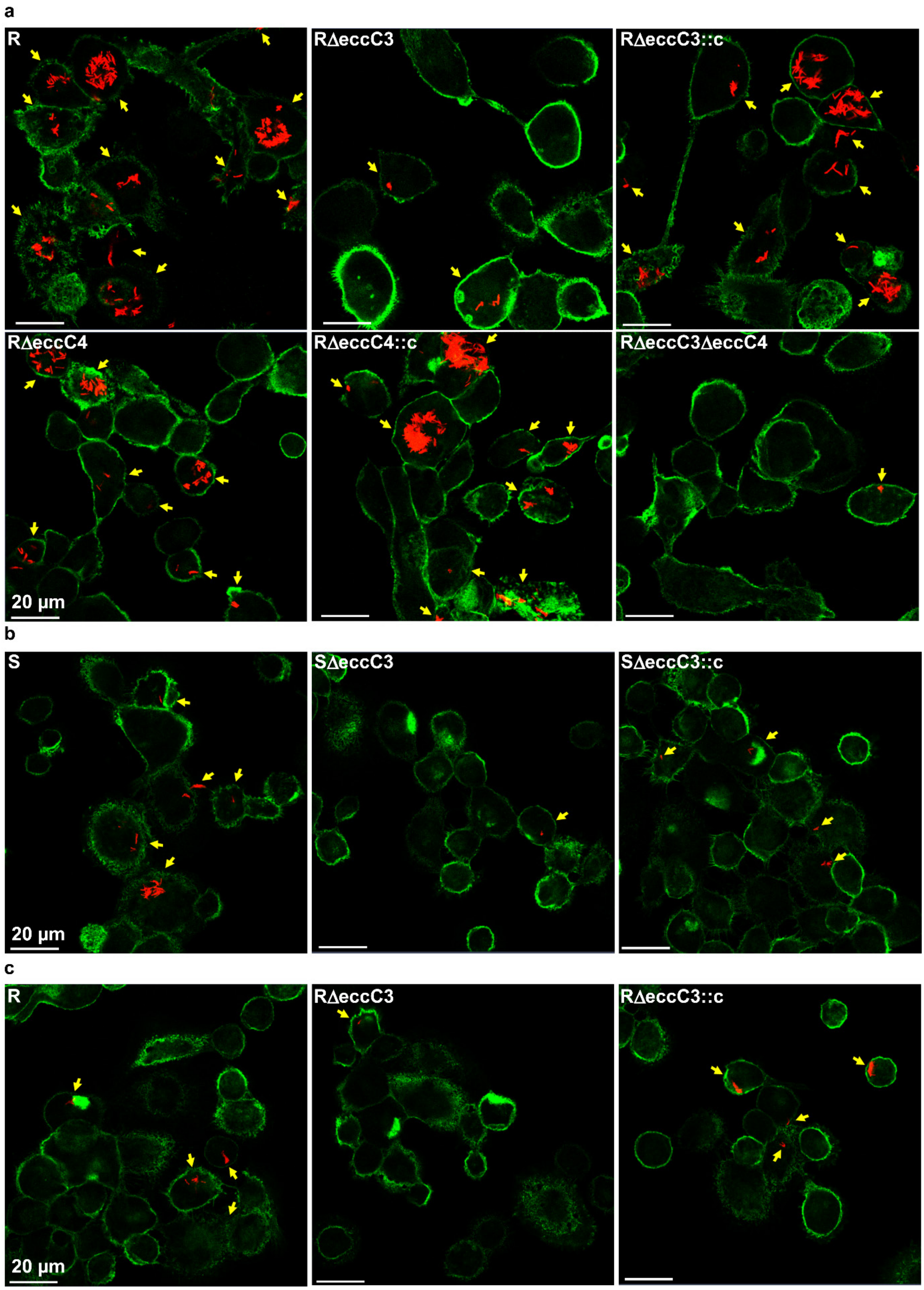

Supplementary Figure 6. EccC3 is crucial for bacilli uptake and like EccC4 is also required for bacterial growth in infected macrophages. (**a**) Immunofluorescent fields taken after 72 hpi of

cells infected with R, RΔ*eccC3*, RΔ*eccC3*::c, RΔ*eccC4*, RΔ*eccC4*::c, and RΔ*eccC3/*Δ*eccC4.* THP-1 macrophages were infected with fluorescent S, SΔ*eccC3*, and SΔ*eccC3*::c strains (**b**) or R, RΔ*eccC3*, and RΔ*eccC3*::c strains (**c**) at a MOI of 2:1. Immunofluorescent fields were taken after 4 hpi at a 40x magnification using a confocal microscope, showing the periphery of the macrophages (green) infected with various *Mab* strains (red). Yellow arrows point to mycobacteria-infected cells. Scale bar, 20 µm.

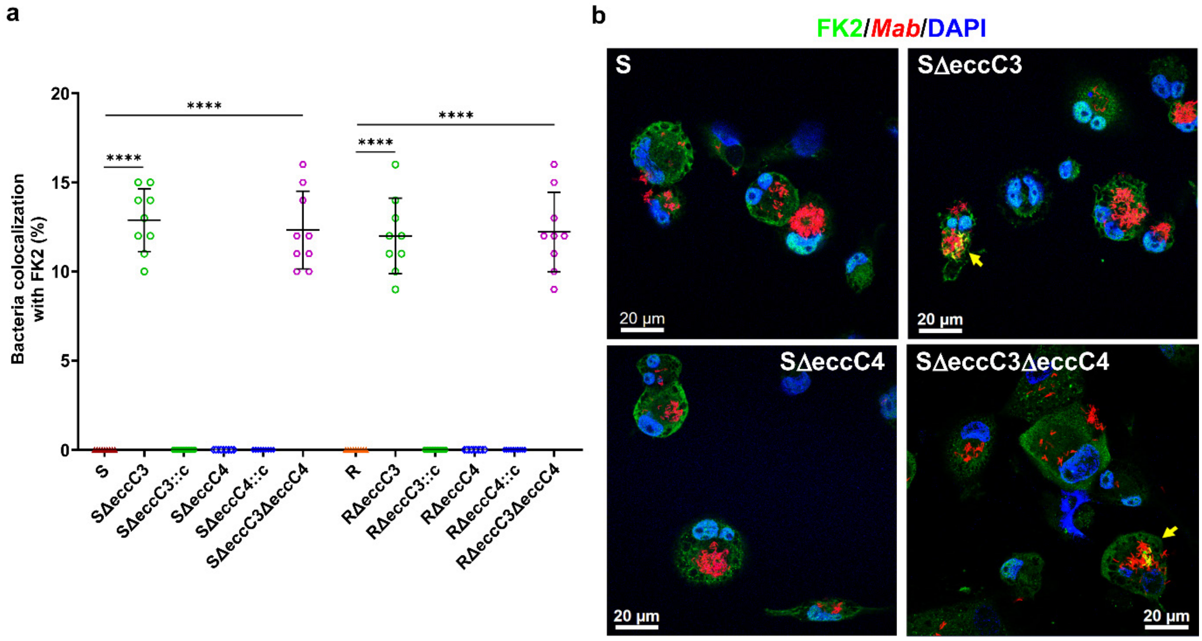

Supplementary Figure 7. Co-localization of intracellular bacteria with FK2 in infected macrophages. (**a**) Percentage of infected macrophages containing at least one positively stained phagosome ($FK2^+$). The values represent the mean of 900 infected cells analyzed from three experiments (n=900). Statistical significance was determined using ANOVA with Tukey's test; ****, *P*<0.0001. (**b**) Fluorescence images of THP-1 cells infected with *Mab* S, SΔ*eccC3*, SΔ*eccC4*, and SΔ*eccC3/*Δ*eccC4*. *Mab* (red), FK2 (green), DAPI (blue). Arrows indicate intracellular mycobacteria co-localizing with FK2. Data are mean ± SD.

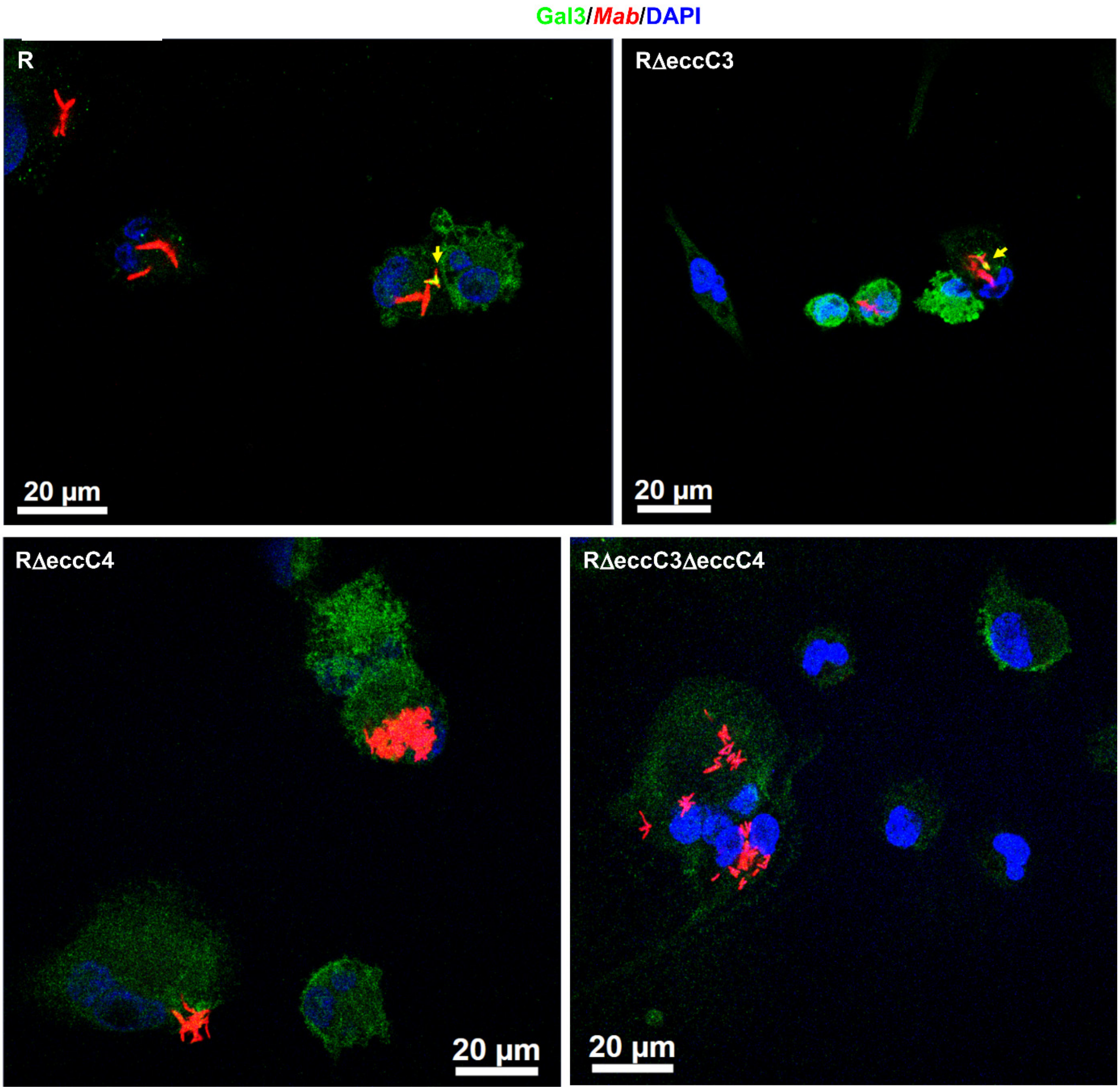

Supplementary Figure 8. ESX-4-dependent phagosomal membrane damage and galectin-3 accumulation in infected macrophages. The recruitment of galectin-3 hinges on the functional ESX-4 system, crucial for bacterial escape from phagosomes. The absence of ESX-4 significantly reduces bacterial escape, thereby preventing the recruitment and accumulation of galectin-3. *Mab* is identified by its autofluorescence when excited at a red wavelength. Representative confocal images show macrophages infected with various fluorescent *Mab* strains (R, RΔ*eccC3*, RΔ*eccC4*, and RΔ*eccC3/*Δ*eccC4*) depicted in red and staining for galectin-3 in green.

Supplementary Table 1. Primers used in this study. Restriction sites are in bold and underlined.

Number	5'→3' sequence	Cloning technique (Restriction site or In-Fusion)	F (sense), R (antisense)
**Cloning in pUX1-*katG***			
1	gaga**TTAATTAA**TCAATCTCGGAGATGTCGCCGTCA	PacI	To clone the left arm of *eccC3* in pUX1-*katG* (F)
2	gaga**CAATTG**GACGATCAGAATCACGATCACGACTGGCAT	MfeI	To clone the left arm of *eccC3* in pUX1-*katG* (R)
3	gaga**CAATTG**ATCTTGTTGTCCGACAACGATGTTCCAACGT	MfeI	To clone the right arm of *eccC3* in pUX1-*katG* (F)
4	gaga**GCTAGC**TCGAAGAAGCTCTCGCTGTCGTCTAG	NheI	To clone the right arm of *eccC3* in pUX1-*katG* (R)
5	gaga**TTAATTAA**GAATCGTCATAGACCCTGTTCGCGAA	PacI	To clone the left arm of *eccC4* in pUX1-*katG* (F)
6	gaga**CAATTG**GACAAAGAAGGCGATCATGCCGACCA	MfeI	To clone the left arm of *eccC4* in pUX1-*katG* (R)
7	gaga**CAATTG**AATAAGGACGAGGGCGTGCTGCT	MfeI	To clone the right arm of *eccC4* in pUX1-*katG* (F)
8	gaga**GCTAGC**ATGAGTTGCACCCTTCCCTCCAC	NheI	To clone the right arm of *eccC4* in pUX1-*katG* (R)
**Primers to verify double homologous recombination**			
9	CAAACCAGCACCGATAACCGCAGCGT	-	To check double homologous recombination Δ*eccC3* (F)
10	TACGCATGAAGTCCTGCAAGATCTTCTG	-	To check double homologous recombination Δ*eccC3* (R)
11	CTGTAAATCGGCGGTTCGAACAGCAC	-	To check double homologous recombination Δ*eccC4* (F)
12	GAAATCGCTGAATGCTCCTTGAGGCT	-	To check double homologous recombination Δ*eccC4* (R)
**Cloning in pMV306-KanR-Hsp60 promoter**			
13	gaga**CAATTG**CCTTGAGCCGTCTCATCTTCGAACCCCAT	MfeI	To clone the *eccC3-HA* ORF in pMV306 (F)
14	gaga**GTTAAC**CTAAGCGTAATCTGGAACATCGTATGGGTATCGTGGGTACTCCCTTCCCTGA	HpaI	To clone the *eccC3-HA* ORF in pMV306 (R)
15	gaga**CAATTG**CCGTGCCGGGTGGCGAGGTCAAT	MfeI	To clone the *eccC4-HA* ORF in pMV306 (F)
16	gaga**GTTAAC**CTAAGCGTAATCTGGAACATCGTATGGGTACTGTGGTTCACTCCAAGCGACCTGGAT	HpaI	To clone the *eccC4-HA* ORF in pMV306 (R)
17	GTTAACTAGCGTACGATCGACTGCCAG	In-Fusion cloning	To amplify the pMV306-*eccC3* or pMV306-*eccC4* vectors (F)
18	CTGTGGTTCACTCCAAGCGACCTGGAT	In-Fusion cloning	To amplify the pMV306-*eccC3* vector (R)
19	TCGTGGGTACTCCCTTCCCTGA	In-Fusion cloning	To amplify the pMV306-*eccC4* vector (R)
20	AGGGAAGGGAGTACCCACGACTgGAGTCgGGcGGTGGTGGcTCgATGGTGAGCAAGGGCGAGGA	In-Fusion cloning	To clone the *mNeonGreen* ORF in pMV306-*eccC3* vector (F)
21	TCGATCGTACGCTAGTTAACTCACTTGTACAGCTCGTCCATGCCCA	In-Fusion cloning	To clone the *mNeonGreen* ORF in pMV306-*eccC3* or pMV306-*eccC4* vectors (R)

22	TCGCTTGGAGTGAACCACAGCTgGAGTCgGG cGGTGGTGGcTCgATGGTGAGCAAGGGCGAGGA	In-Fusion cloning	To clone the *mNeonGreen* ORF in pMV306-*eccC4* vector (F)
23	gaga<u>**CAATTG**</u>CCATGAGTTTGCTTGACGCACACAT TCCC	MfeI	To clone the *esxG/esxH-HA* genes into the pMV306 vector (F)
24	gaga<u>**GTTAAC**</u>CTAAGCGTAATCTGGAACATC GTATGGGTATGCGCCCCACTTGGCTCCTT	HpaI	To clone the *esxG/esxH-HA* genes into the pMV306 vector (R)
25	gaga<u>**CAATTG**</u>CCATGAGTTTGCTTGATGCGC ATATTCCGGCTTT	MfeI	To clone the *esxG2/esxH2-HA* genes into the pMV306 vector (F)
26	gaga<u>**GTTAAC**</u>CTAAGCGTAATCTGGAACATCGTAT GGGTATGCGCCCCACTTGGCTCCTTC	HpaI	To clone the *esxG2/esxH2-HA* genes into the pMV306 vector (R)
27	gaga<u>**CAATTG**</u>GGGTGTCGGCTCCGATTTGGATGG	MfeI	To clone the *ppe4-HA* gene into the pMV306 vector (F)
28	gaga<u>**GTTAAC**</u>TCAAGCGTAATCTGGAAC ATCGTATGGGTAGGTGCTCGCCCCCCTTTC	HpaI	To clone the *ppe4-HA* gene into the pMV306 vector (R)
29	GTGTCGGCTCCGATTTGGATGGCTT	In-Fusion cloning	To amplify the pMV306-*ppe4-HA* vector (F)
30	CCCAATTGTCTTGGCCATTGCGAAGT	In-Fusion cloning	To amplify the pMV306-*ppe4-HA* vector (R)
31	CAATGGCCAAGACAATTGGGATGAA TCTCAACGTCGTTCCGGAAGGT	In-Fusion cloning	To clone the *pe5* ORF in pMV306-*ppe4-HA* vector (F)
32	ATCCAAATCGGAGCCGACACCGGTGTTAACCCCGC GCGAT	In-Fusion cloning	To clone the *pe5* ORF in pMV306-*ppe4-HA* vector (R)
33	GTTAACTAGCGTACGATCGACTGCCAG	In-Fusion cloning	To amplify the pMV306-*pe5-ppe4-HA* vector (F)
34	TTAAGCGTAATCTGGAACATCGTATGGGTAGGT	In-Fusion cloning	To amplify the pMV306-*pe5-ppe4-HA* vector (R)
35	ATGTTCCAGATTACGCTTAAACGCCCTTGGTGCTC ATGCACCT	In-Fusion cloning	To clone the *espG3-strep* ORF in pMV306-*pe5-ppe4-HA* vector (F)
36	TCGATCGTACGCTAGTTAACTCACTTCTCGAACTG CG GGTGGCTCCAATCGAAGTCTCGGGTCACGCGG	In-Fusion cloning	To clone the *espG3-strep* ORF in pMV306-*pe5-ppe4-HA* vector (R)
37	gaga<u>**CAATTG**</u>CCATGAGTCTCAATGTCGTTCCCGA GGG	MfeI	To clone the *MAB_0664-HA* ORF in pMV306 (F)
38	gaga<u>**GTTAAC**</u>CTAAGCGTAATCTGGAACATCGTAT G GGTATGCGATTCCCGGTAGATACGTTGCAG	HpaI	To clone the *MAB_0664-HA* ORF in pMV306 (R)
39	gaga<u>**CAATTG**</u>CCATGAATCTCAACGTCGTTCCGGA AGGTCT	MfeI	To clone the *MAB_0046-MAB_0047-HA* genes into the pMV306 vector (F)
40	gaga<u>**GTTAAC**</u>CTAAGCGTAATCTGGAACATCGTAT GGGTA	HpaI	To clone the *MAB_0046-MAB_0047-HA* genes into the pMV306 vector (R)
41	gaga<u>**CAATTG**</u>CCGTGGCTGTTTTTCAGAATGACCT GGC	MfeI	To clone the *esxUT-HA* ORFs in pMV306 (F)
42	gaga<u>**AAGCTT**</u>CTAAGCGTAATCTGGAACATCGTAT GG GTAGTGGTGCCAGGCGCCGG	HindIII	To clone the *esxUT-HA* ORFs in pMV306 (R)
43	gaga<u>**GCTAGC**</u>AGGTGACCACAACGACGCGCC	NheI	To clone the: *esxG/esxH-HA* or pe5/ppe4-HA/espG3-strep or *esxG2/esxH2-HA* or

			MAB_0664-HA or *MAB_0046-MAB_0047-HA* ORFs in pMV306-*eccC3-mNeonGreen* vector (F) and *esxU/esxT-HA* gene into the pMV306-*eccC4-mNeonGreen* vector (F)
44	gaga**GCTAGC**CTAAGCGTAATCTGGAACATCGTATGGG	NheI	To clone the: *esxG/esxH-HA* or *esxG2/esxH2-HA* or *MAB_0664-HA* or *MAB_0046-MAB_0047-HA* ORFs in pMV306-*eccC3-mNeonGreen* vector (R) and *esxU/esxT-HA* gene into the pMV306-*eccC4-mNeonGreen* vector (R)
45	gaga**GCTAGC**TCACTTCTCGAACTGCGGGTGGCT	NheI	To clone the pe5/ppe4-HA/espG3-strep genes into the pMV306-*eccC3-mNeonGreen* vector (R)

127

Supplementary Table 2. Strains used in this study.

Name	Description/genotype	Resistance	Reference
M. abscessus Smooth (S)	*M. abscessus* sensu stricto, strain CIP104536^{T}, S morphotype	-	Laboratoire de Référence des Mycobactéries
M. abscessus Rough (R)	*M. abscessus* sensu stricto, strain CIP104536^{T}, R morphotype	-	Laboratoire de Référence des Mycobactéries
Δ3	Unmarked deletion of *eccC3* (*MAB_2232c*) in *M. abscessus*	-	This study
Δ4	Unmarked deletion of *eccC4* (*MAB_3756c*) in *M. abscessus*	-	This study
Δ34	Unmarked deletion of *eccC3* (*MAB_2232c*) and *eccC4* (*MAB_3756c*) in *M. abscessus*	-	This study
Δ3 + pMV306-*eccC3-HA*	*ΔeccC3* carrying pMV306-*eccC3-HA*	Kan	This study
Δ4 + pMV306-*eccC4-HA*	*ΔeccC4* carrying pMV306-*eccC4-HA*	Kan	This study
Δ3 + pMV306-*eccC3-mNeonGreen*	*ΔeccC3* carrying pMV306-*eccC3-mNeonGreen*	Kan	This study
Δ4 + pMV306-*eccC4-mNeonGreen*	*ΔeccC4* carrying pMV306-*eccC4-mNeonGreen*	Kan	This study
S + pMV306-*MAB_2229c/MAB_2228c-HA*	*S* carrying pMV306-*MAB_2229c/MAB_2228c-HA*	Kan	This study
Δ3 + pMV306-*MAB_2229c/MAB_2228c-HA*	*Δ3* carrying pMV306-*MAB_2229c/MAB_2228c-HA*	Kan	This study
Δ4 + pMV306-*MAB_2229c/MAB_2228c-HA*	*Δ4* carrying pMV306-*MAB_2229c/MAB_2228c-HA*	Kan	This study
Δ34 + pMV306-*MAB_2229c/MAB_2228c-HA*	*Δ34* carrying pMV306-*MAB_2229c/MAB_2228c-HA*	Kan	This study
S + pMV306-*MAB_2231c/MAB_2230c-HA/MAB_2227c-Strep*	*S* carrying pMV306-*MAB_2231c/MAB_2230c-HA/MAB_2227c-Strep*	Kan	This study

Δ3 + pMV306-*MAB_2231c/MAB_2230c-HA/MAB_2227c-Strep*	*Δ3* carrying pMV306-*MAB_2231c/MAB_2230c-HA/MAB_2227c-Strep*	Kan	This study
Δ4 + pMV306-*MAB_2231c/MAB_2230c-HA/MAB_2227c-Strep*	*Δ4* carrying pMV306-*MAB_2231c/MAB_2230c-HA/MAB_2227c-Strep*	Kan	This study
Δ34 + pMV306-*MAB_2231c/MAB_2230c-HA/MAB_2227c-Strep*	*Δ34* carrying pMV306-*MAB_2231c/MAB_2230c-HA/MAB_2227c-Strep*	Kan	This study
S + pMV306-*MAB_3754c/MAB_3753c-HA*	*S* carrying pMV306-*MAB_3754c/MAB_3753c-HA*	Kan	This study
Δ3 + pMV306-*MAB_3754c/MAB_3753c-HA*	*Δ3* carrying pMV306-*MAB_3754c/MAB_3753c-HA*	Kan	This study
Δ4 + pMV306-*MAB_3754c/MAB_3753c-HA*	*Δ4* carrying pMV306-*MAB_3754c/MAB_3753c-HA*	Kan	This study
Δ34 + pMV306-*MAB_3754c/MAB_3753c-HA*	*Δ34* carrying pMV306-*MAB_3754c/MAB_3753c-HA*	Kan	This study
S + pMV306-*MAB_0664-HA*	*S* carrying pMV306-*MAB_0664-HA*	Kan	This study
Δ3 + pMV306-*MAB_0664-HA*	*Δ3* carrying pMV306-*MAB_0664-HA*	Kan	This study
Δ4 + pMV306-*MAB_0664-HA*	*Δ4* carrying pMV306-*MAB_0664-HA*	Kan	This study
Δ34 + pMV306-*MAB_0664-HA*	*Δ34* carrying pMV306-*MAB_0664-HA*	Kan	This study
S + pMV306-*MAB_0665/MAB_0666-HA*	*S* carrying pMV306-*MAB_0665/MAB_0666-HA*	Kan	This study
Δ3 + pMV306-*MAB_0665/MAB_0666-HA*	*Δ3* carrying pMV306-*MAB_0665/MAB_0666-HA*	Kan	This study
Δ4 + pMV306-*MAB_0665/MAB_0666-HA*	*Δ4* carrying pMV306-*MAB_0665/MAB_0666-HA*	Kan	This study
Δ34 + pMV306-*MAB_0665/MAB_0666-HA*	*Δ34* carrying pMV306-*MAB_0665/MAB_0666-HA*	Kan	This study
S + pMV306-*MAB_0046/MAB_0047-HA*	*S* carrying pMV306-*MAB_0046/MAB_0047-HA*	Kan	This study

Δ3 + pMV306-*MAB_0046/MAB_0047-HA*	*Δ3* carrying pMV306-*MAB_0046/MAB_0047-HA*	Kan	This study
Δ4 + pMV306-*MAB_0046/MAB_0047-HA*	*Δ4* carrying pMV306-*MAB_0046/MAB_0047-HA*	Kan	This study
Δ34 + pMV306-*MAB_0046/MAB_0047-HA*	*Δ34* carrying pMV306-*MAB_0046/MAB_0047-HA*	Kan	This study
Δ3 + pMV306-*eccC3-mNeonGreen-MAB_2229c/MAB_2228c-HA*	*Δ3* carrying pMV306-*eccC3-mNeonGreen-MAB_2229c/MAB_2228c-HA*	Kan	This study
Δ3 + pMV306- *eccC3-mNeonGreen-MAB_2231c/MAB_2230c-HA/MAB_2227c-Strep*	*Δ3* carrying pMV306-*eccC3-mNeonGreen-MAB_2231c/MAB_2230c-HA/MAB_2227c-Strep*	Kan	This study
Δ3 + pMV306-*eccC3-mNeonGreen-MAB_0665/MAB_0666-HA*	*Δ3* carrying pMV306-*eccC3-mNeonGreen-MAB_0665/MAB_0666-HA*	Kan	This study
Δ3 + pMV306-*eccC3-mNeonGreen-MAB_0664-HA*	*Δ3* carrying pMV306-*eccC3-mNeonGreen-MAB_0664-HA*	Kan	This study
Δ3 + pMV306-*eccC3-mNeonGreen-MAB_0046/MAB_0047-HA*	*Δ3* carrying pMV306-*eccC3-mNeonGreen-MAB_0046/MAB_0047-HA*	Kan	This study
Δ4 + pMV306-*eccC4-mNeonGreen-MAB_3754c/MAB_3753c-HA*	*Δ4* carrying pMV306-*eccC4-mNeonGreen-MAB_3754c/MAB_3753c-HA*	Kan	This study

129 Kan, kanamycin

Supplementary Table 3. Plasmids used in this study.

**Plasmids**	**Description**	**Resistance**	**Ref**
pTEC27	*tdTomato* expressed under the control of a strong mycobacterial promoter by a multicopy *E. coli*/mycobacterial shuttle vector	Hyg	[1]
pMV306	Integrative vector	Kan	[2]
pMV306-mScarlet	pMV306 allowing expression of mScarlet in mycobacteria	Kan	[3]
pUX1-*katG*	A pUX1 variant including *katG* gene of *M. tuberculosis* as a marker to counter-select in the presence of isoniazid and allowing to generate unmarked chromosomal alterations.	Kan, INH	[4]
eccC3 pUX1-katG	pUX1-*katG* including the upstream and downstream sequences around *eccC3*	Kan, INH	This study
eccC4 pUX1-katG	pUX1-*katG* including the upstream and downstream sequences around *eccC4*	Kan, INH	This study
pMV306-*eccC3-HA*	pMV306 enabling the expression of EccC3-HA in mycobacteria	Kan	This study
pMV306-*eccC4-HA*	pMV306 enabling the expression of EccC4-HA in mycobacteria	Kan	This study
pMV306-*eccC3-mNeonGreen*	pMV306 enabling the expression of EccC3-mNeonGreen in mycobacteria	Kan	This study
pMV306-*eccC4-mNeonGreen*	pMV306 enabling the expression of EccC3-mNeonGreen in mycobacteria	Kan	This study
pMV306-*MAB_2229c/MAB_2228c-HA*	pMV306 enabling the expression of EsxG/EsxH-HA in mycobacteria	Kan	This study
pMV306-*MAB_2231c/MAB_2230c-HA/MAB_2227c-Strep*	pMV306 enabling the expression of PE5/PPE4-HA/EspG3-Strep in mycobacteria	Kan	This study
pMV306-*MAB_3754c/MAB_3753c-HA*	pMV306 enabling the expression of EsxU/EsxT-HA in mycobacteria	Kan	This study
pMV306-*MAB_0664-HA*	pMV306 enabling the expression of MAB_0664-HA in mycobacteria	Kan	This study
pMV306-*MAB_0665/MAB_0666-HA*	pMV306 enabling the expression of EsxG2/EsxH2-HA in mycobacteria	Kan	This study

pMV306-*MAB_0046/MAB_0047-HA*	pMV306 enabling the expression of MAB_0046/MAB_0047-HA in mycobacteria	Kan	This study
pMV306-*eccC3-mNeonGreen-MAB_2229c/MAB_2228c-HA*	pMV306 enabling the expression of both EccC3-mNeonGreen and MAB_2229c/MAB_2228c-HA in mycobacteria	Kan	This study
pMV306-*eccC3-mNeonGreen-MAB_2231c/MAB_2230c-HA/MAB_2227c-strep*	pMV306 enabling the expression of both EccC3-mNeonGreen and PE5/PPE4-HA/EspG3-Strep in mycobacteria	Kan	This study
pMV306-*eccC3-mNeonGreen-MAB_0665/MAB_0666-HA*	pMV306 enabling the expression of both EccC3-mNeonGreen and MAB_0665/MAB_0666-HA in mycobacteria	Kan	This study
pMV306-*eccC3-mNeonGreen-MAB_0664-HA*	pMV306 enabling the expression of both EccC3-mNeonGreen and MAB_0664-HA in mycobacteria	Kan	This study
pMV306-*eccC3-mNeonGreen-MAB_0046/MAB_0047-HA*	pMV306 enabling the expression of both EccC3-mNeonGreen and MAB_0046/MAB_0047-HA in mycobacteria	Kan	This study
pMV306-*eccC4-mNeonGreen-MAB_3754c/MAB_3753c-HA*	pMV306 enabling the expression of both EccC4-mNeonGreen and MAB_3754c/MAB_3753c-HA in mycobacteria	Kan	This study

Hyg, hygromycin; Kan, kanamycin; INH, isoniazid

1. Takaki, K., Davis, J. M., Winglee, K. & Ramakrishnan, L. Evaluation of the pathogenesis and treatment of *Mycobacterium marinum* infection in zebrafish. *Nat Protoc* **8**, 1114–1124 (2013).
2. Stover, C. K. *et al.* New use of BCG for recombinant vaccines. *Nature* **351**, 456–460 (1991).
3. Pichler, V. *et al.* The diversity of clinical *Mycobacterium abscessus* isolates in morphology, glycopeptidolipids and infection rates in a macrophage model. *J Med Microbiol* **73**, 001869 (2024).
4. Daher, W. *et al.* Glycopeptidolipid glycosylation controls surface properties and pathogenicity in *Mycobacterium abscessus*. *Cell Chem Biol* **29**, 910-924.e7 (2022).

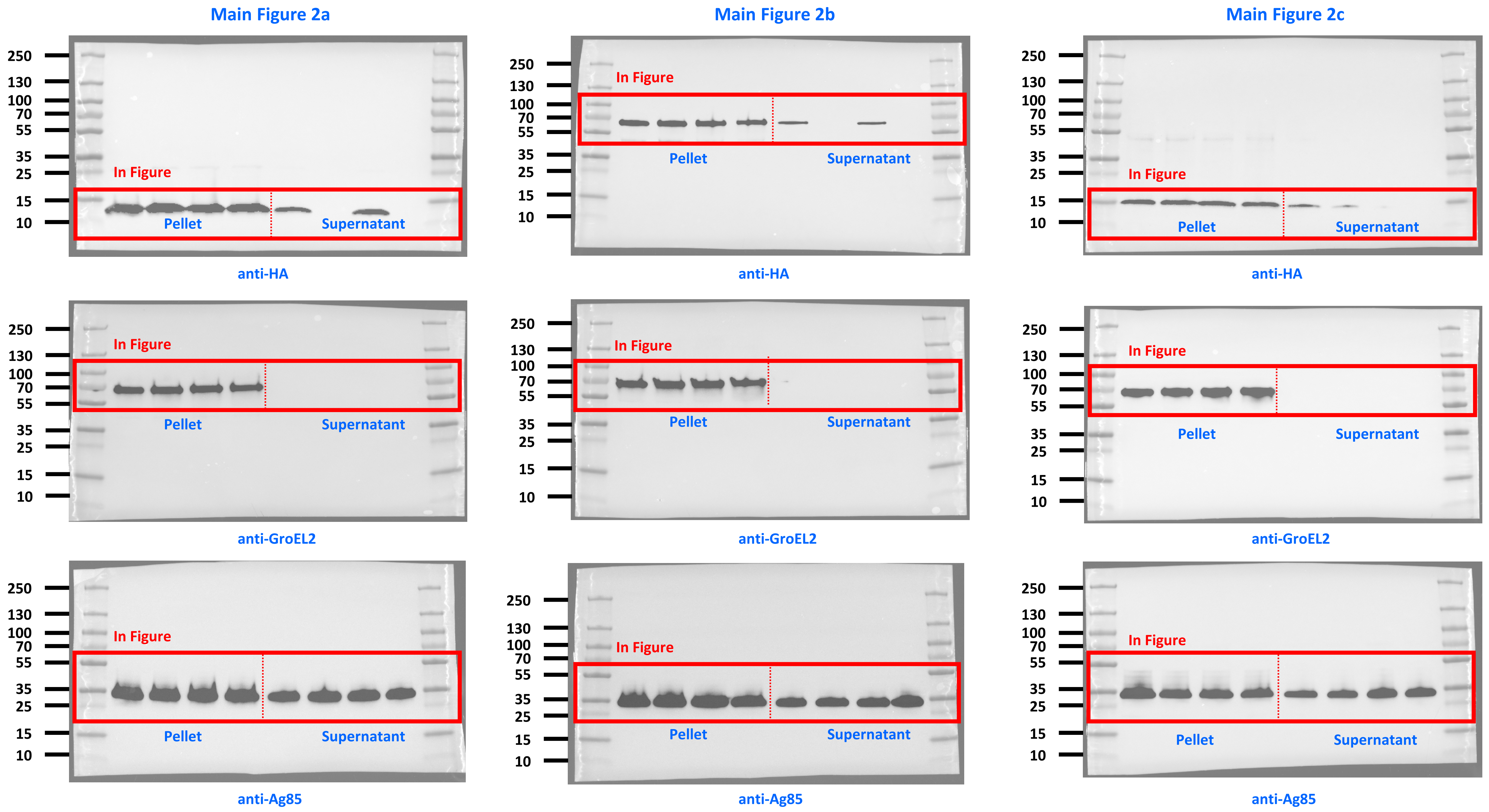

Uncropped western blots from main figures

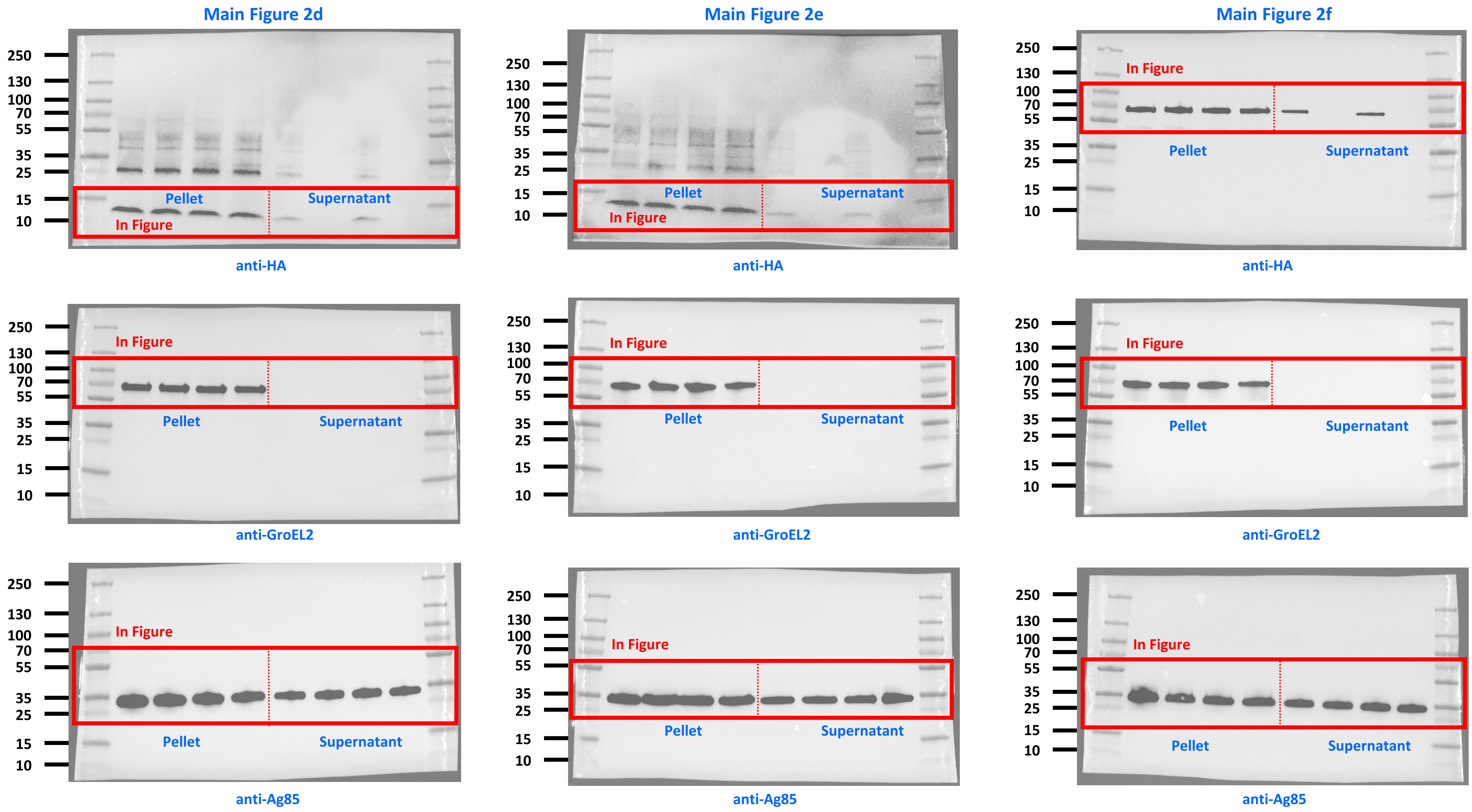

Uncropped western blots from main figures

Main Figure 2h

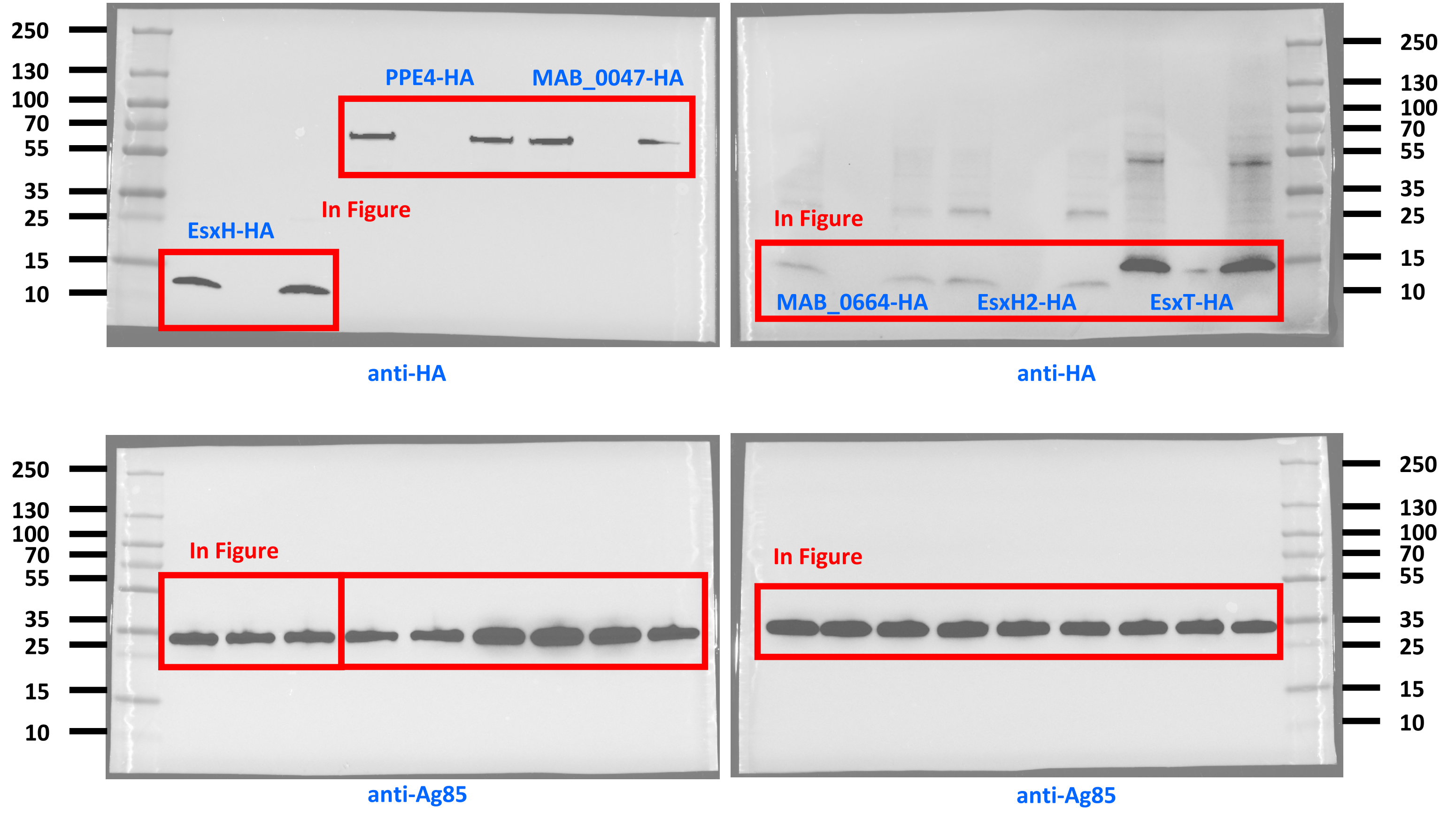

Main Figure 3c

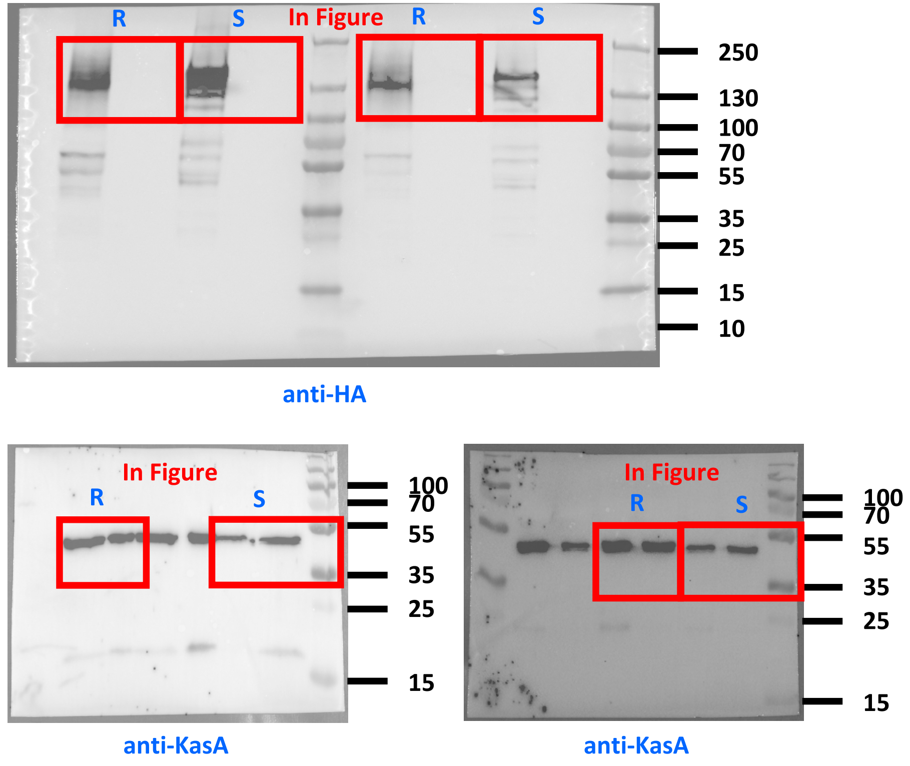

Uncropped western blots from main figures

Main Figure 3d

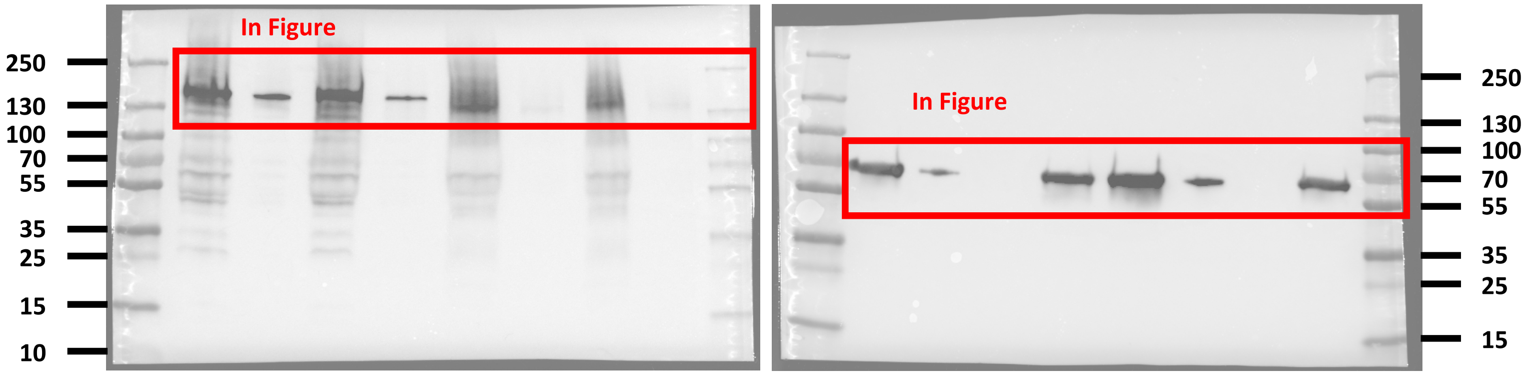

anti-HA

anti-GroEL2

Main Figure 3e

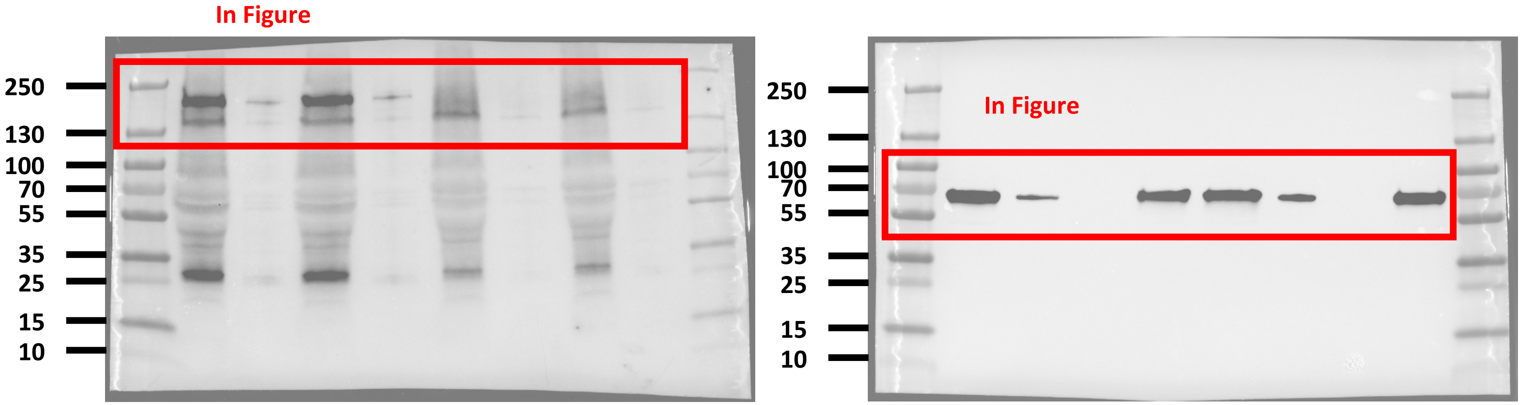

anti-mNeonGreen

anti-GroEL2

Uncropped western blots from main figures

Supplementary Figure 4a

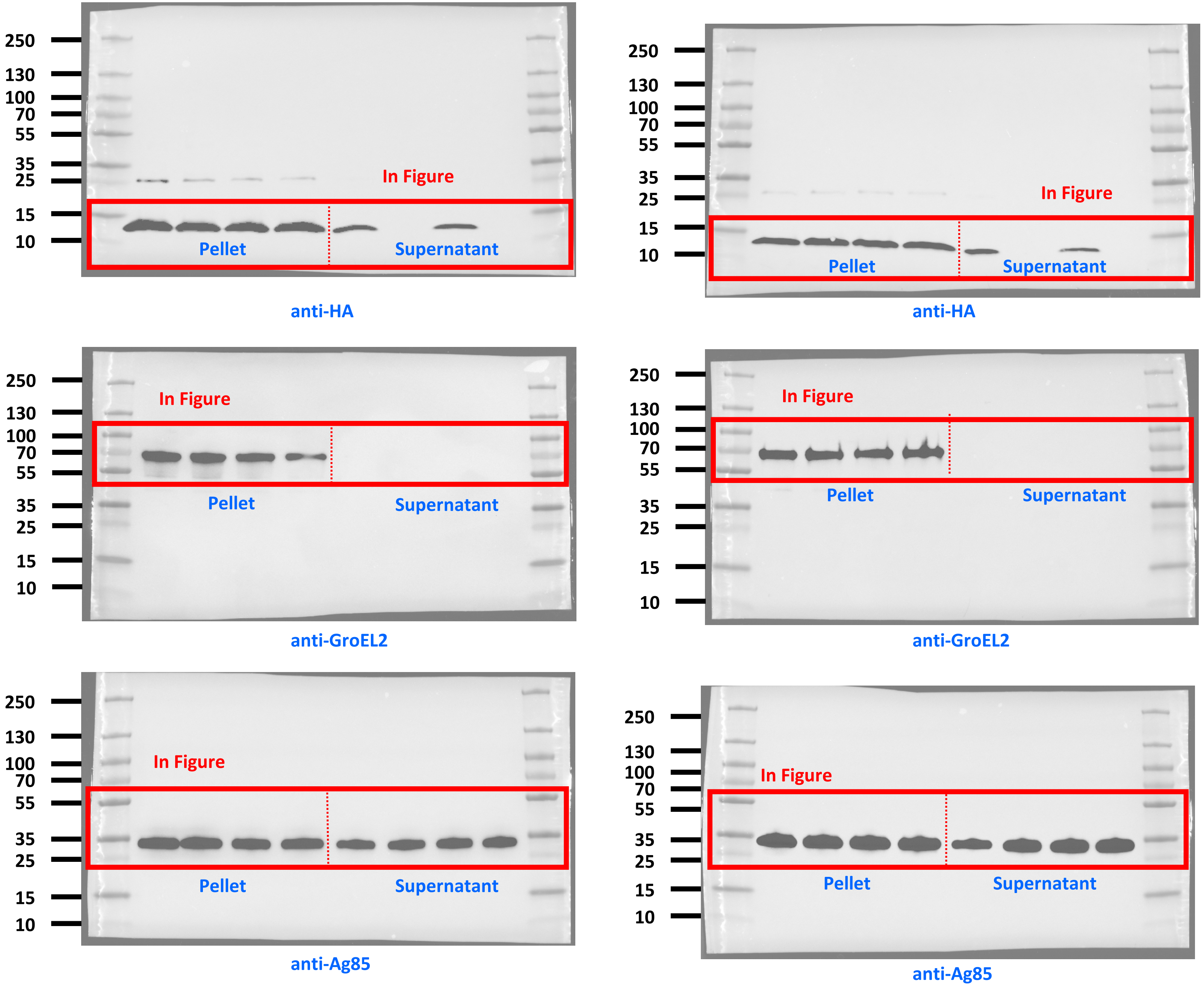

Uncropped western blots from supplementary figures

Supplementary Figure 4b

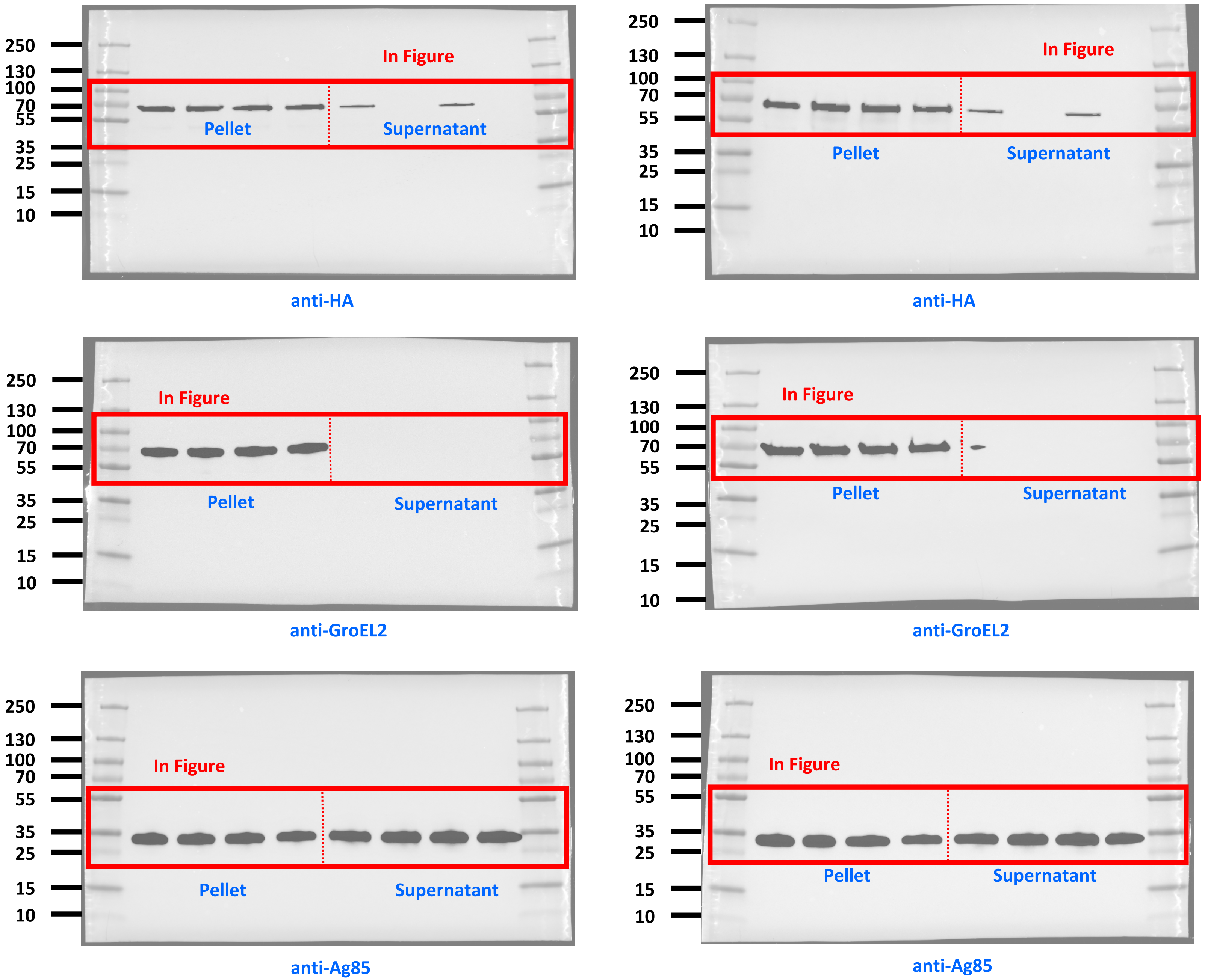

Uncropped western blots from supplementary figures

Supplementary Figure 4c

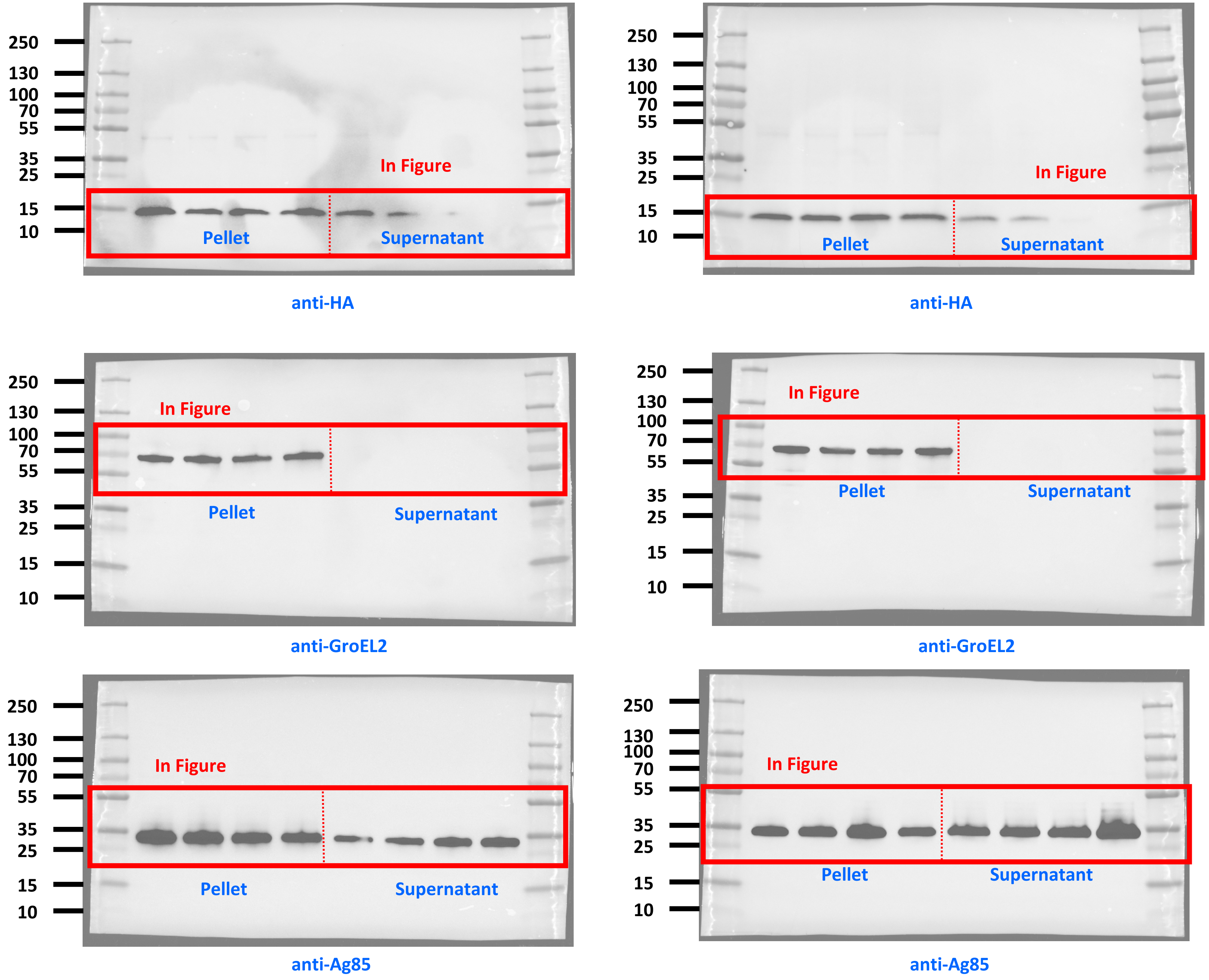

Uncropped western blots from supplementary figures

Supplementary Figure 4d

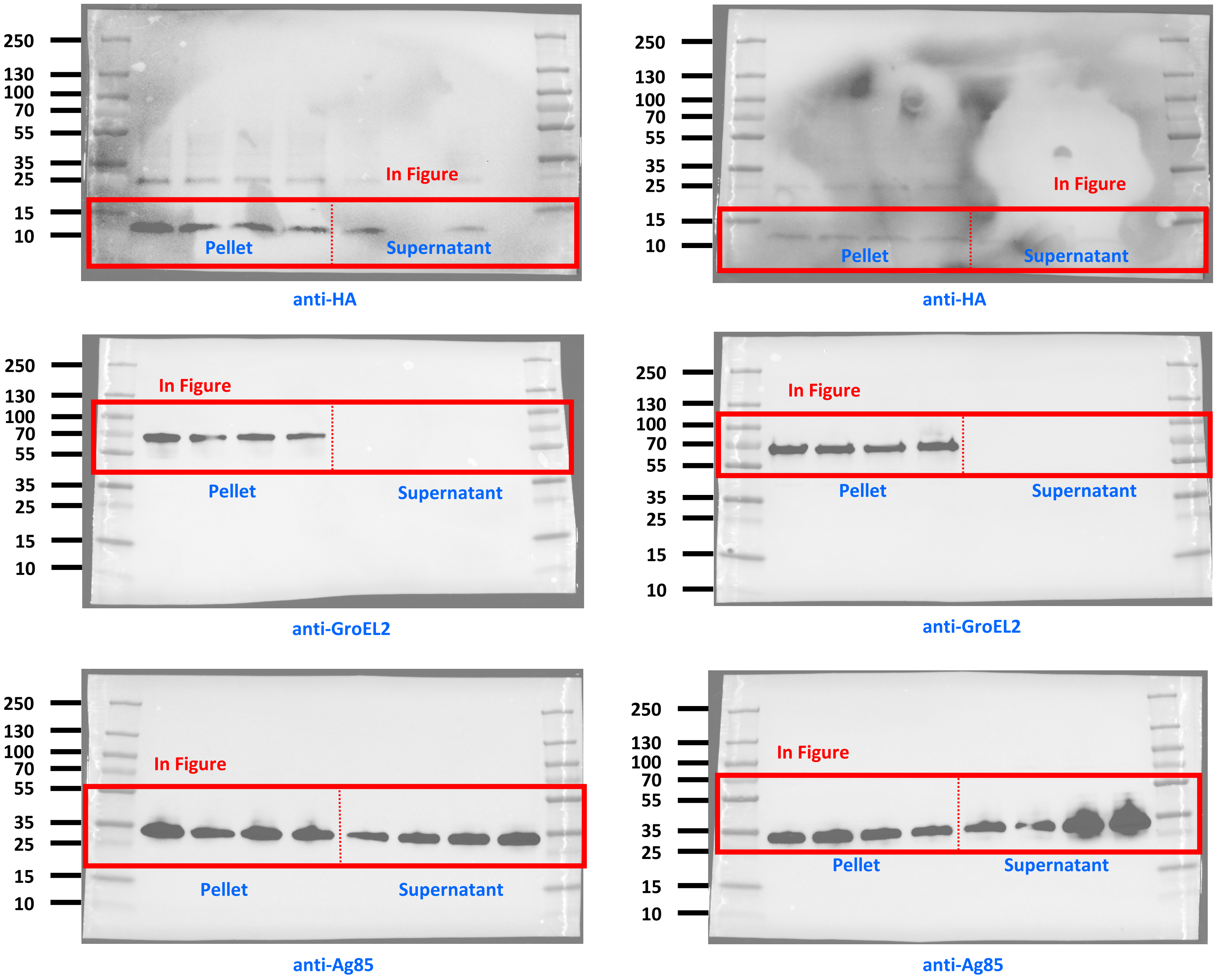

Uncropped western blots from supplementary figures

Supplementary Figure 4e

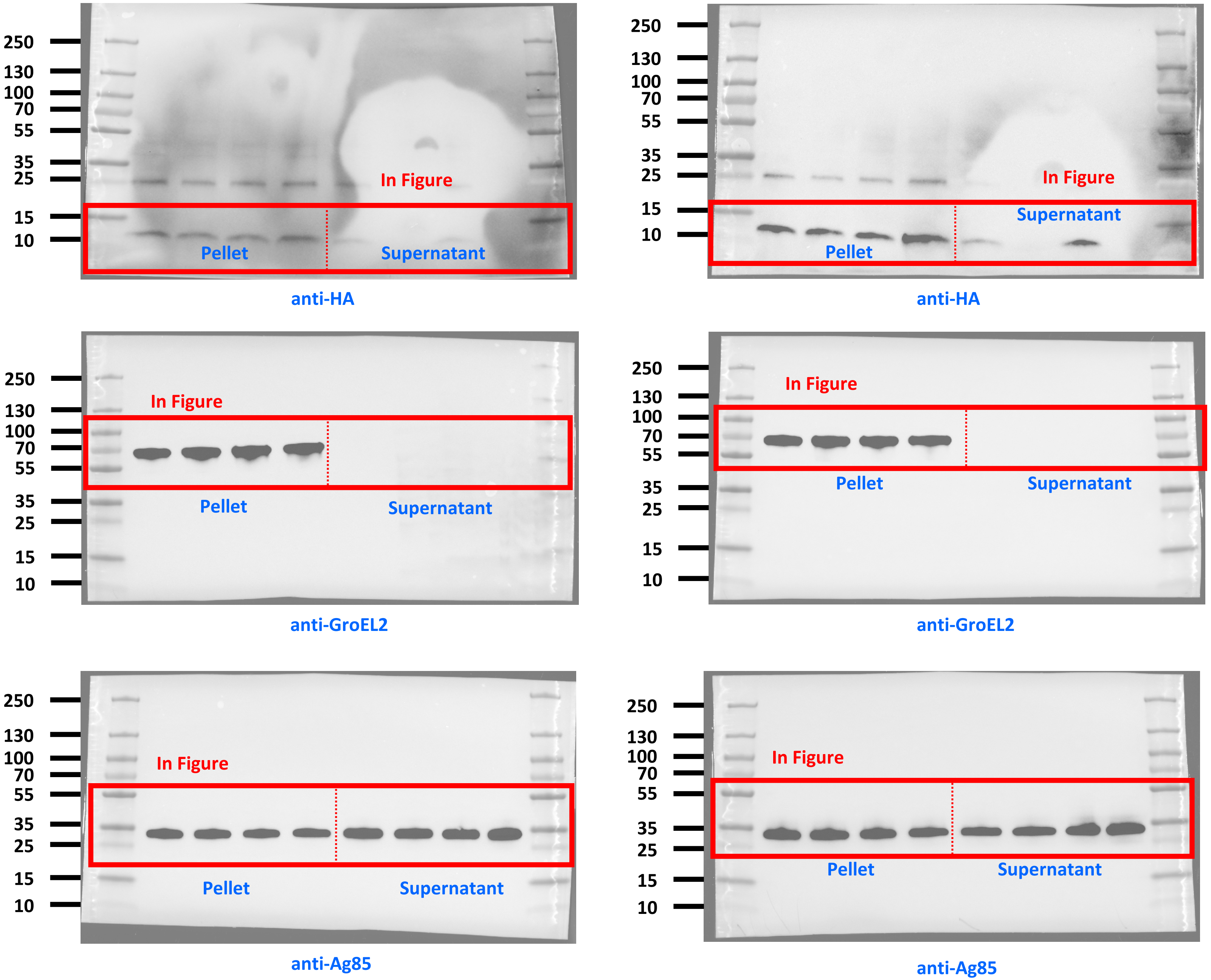

Uncropped western blots from supplementary figures

Supplementary Figure 4f

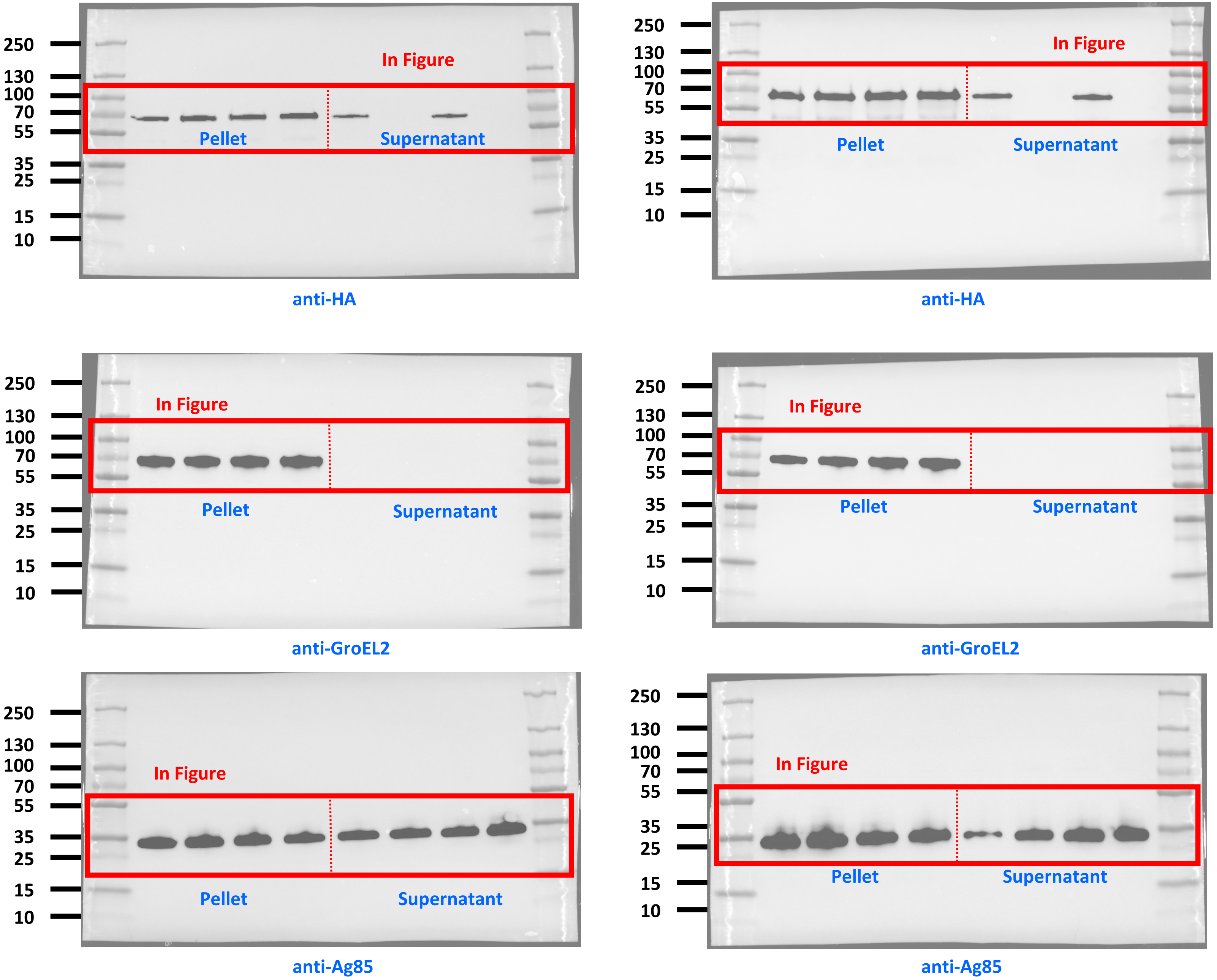

Uncropped western blots from supplementary figures
