# Supplementary material for: Deletion of ESX-3 and ESX-4 secretion systems in Mycobacterium abscessus results in highly impaired pathogenicity
Source: Commun Biol. 2025 Feb 3;8:166. doi: 10.1038/s42003-025-07572-4 (PMC11791044; doi:10.1038/s42003-025-07572-4)
Supplement: Supplementary file 3 — Description of Additional Supplementary File [file 42003_2025_7572_MOESM3_ESM.pdf]

Description of additional supplementary data

File name: Supplementary Data 1

Description: The source data behind the graphs in the paper.
