# Supplementary material for: Deletion of ESX-3 and ESX-4 secretion systems in Mycobacterium abscessus results in highly impaired pathogenicity
Source: Commun Biol. 2025 Feb 3;8:166. doi: 10.1038/s42003-025-07572-4 (PMC11791044; doi:10.1038/s42003-025-07572-4)
Supplement: Supplementary file 4 — Reporting summary [file 42003_2025_7572_MOESM4_ESM.pdf]

nature portfolio

Corresponding author(s): Wassim DAHER, Laurent KREMER

Last updated by author(s): Sep 5, 2024

Reporting Summary

Nature Portfolio wishes to improve the reproducibility of the work that we publish. This form provides structure for consistency and transparency in reporting. For further information on Nature Portfolio policies, see our Editorial Policies and the Editorial Policy Checklist.

Statistics

For all statistical analyses, confirm that the following items are present in the figure legend, table legend, main text, or Methods section.

n/a	Confirmed	
☐	☒	The exact sample size (*n*) for each experimental group/condition, given as a discrete number and unit of measurement
☐	☒	A statement on whether measurements were taken from distinct samples or whether the same sample was measured repeatedly
☐	☒	The statistical test(s) used AND whether they are one- or two-sided<br>*Only common tests should be described solely by name; describe more complex techniques in the Methods section.*
☒	☐	A description of all covariates tested
☒	☐	A description of any assumptions or corrections, such as tests of normality and adjustment for multiple comparisons
☐	☒	A full description of the statistical parameters including central tendency (e.g. means) or other basic estimates (e.g. regression coefficient) AND variation (e.g. standard deviation) or associated estimates of uncertainty (e.g. confidence intervals)
☐	☒	For null hypothesis testing, the test statistic (e.g. *F*, *t*, *r*) with confidence intervals, effect sizes, degrees of freedom and *P* value noted<br>*Give P values as exact values whenever suitable.*
☒	☐	For Bayesian analysis, information on the choice of priors and Markov chain Monte Carlo settings
☒	☐	For hierarchical and complex designs, identification of the appropriate level for tests and full reporting of outcomes
☒	☐	Estimates of effect sizes (e.g. Cohen's *d*, Pearson's *r*), indicating how they were calculated

Our web collection on statistics for biologists contains articles on many of the points above.

Software and code

Policy information about availability of computer code

Data collection	No software was used
Data analysis	Fluorescent images were analyzed with Zen (black edition). Graphs were generated and infection data analyzed with GraphPad Prism 10.1.

For manuscripts utilizing custom algorithms or software that are central to the research but not yet described in published literature, software must be made available to editors and reviewers. We strongly encourage code deposition in a community repository (e.g. GitHub). See the Nature Portfolio guidelines for submitting code & software for further information.

Data

Policy information about availability of data

All manuscripts must include a data availability statement. This statement should provide the following information, where applicable:

- Accession codes, unique identifiers, or web links for publicly available datasets
- A description of any restrictions on data availability
- For clinical datasets or third party data, please ensure that the statement adheres to our policy

The data that support the findings of this study are available from the corresponding authors upon reasonable request.

Research involving human participants, their data, or biological material

Policy information about studies with human participants or human data. See also policy information about sex, gender (identity/presentation), and sexual orientation and race, ethnicity and racism.

Reporting on sex and gender	N/A
Reporting on race, ethnicity, or other socially relevant groupings	N/A
Population characteristics	N/A
Recruitment	N/A
Ethics oversight	N/A

Note that full information on the approval of the study protocol must also be provided in the manuscript.

Field-specific reporting

Please select the one below that is the best fit for your research. If you are not sure, read the appropriate sections before making your selection.

☒ Life sciences ☐ Behavioural & social sciences ☐ Ecological, evolutionary & environmental sciences

For a reference copy of the document with all sections, see nature.com/documents/nr-reporting-summary-flat.pdf

Life sciences study design

All studies must disclose on these points even when the disclosure is negative.

Sample size	N/A
Data exclusions	No data exclusions.
Replication	All experiments were performed in duplicate or triplicate, and performed at least twice. Similar results were found every time.
Randomization	N/A
Blinding	N/A

Reporting for specific materials, systems and methods

We require information from authors about some types of materials, experimental systems and methods used in many studies. Here, indicate whether each material, system or method listed is relevant to your study. If you are not sure if a list item applies to your research, read the appropriate section before selecting a response.

Materials & experimental systems

n/a	Involved in the study	
☐	☒	Antibodies
☐	☒	Eukaryotic cell lines
☒	☐	Palaeontology and archaeology
☐	☒	Animals and other organisms
☒	☐	Clinical data
☒	☐	Dual use research of concern
☒	☐	Plants

Methods

n/a	Involved in the study	
☒	☐	ChIP-seq
☒	☐	Flow cytometry
☒	☐	MRI-based neuroimaging

Antibodies

Antibodies used	Rat anti-HA (3F10) Merck Cat#11867423001, mice anti-Ag85 (Kremer et al., 2002), mice anti-Strep (GT661) SIGMA Cat#SAB2702216, rat anti-KasA (Kremer et al., 2002)
Validation	Anti-HA, High Affinity recognizes the 9-amino acid sequence YPYDVPDYA, derived from the human influenza hemagglutinin (HA)

Validation	protein; Anti-HA High Affinity antibody is used for the detection of native influenza hemagglutinin protein and recombinant proteins that contain the HA epitope using: Dot blots, ELISA, Immunocytochemistry, Immunoprecipitation, Western blots.<br>Monoclonal Anti-Strep Tag antibody produced in mouse clone GT661, affinity isolated antibody. Anti-Strep; high affinity recognizes the 9-amino acid sequence AWRHPQFGG. Suitable for immunoprecipitation (IP), indirect immunofluorescence, western blot: 1000-10000.

Eukaryotic cell lines

Policy information about cell lines and Sex and Gender in Research

Cell line source(s)	THP-1 human macrophage line.
Authentication	Supplied by: European Collection of Authenticated Cell Cultures (ECACC), Catalogue No.: 88081201.
Mycoplasma contamination	I confirm that THP-1 cells are not contaminated with mycoplasma on the basis of DAPI labeling which revealed no punctate staining under microscopy.
Commonly misidentified lines<br>(See ICLAC register)	N/A

Animals and other research organisms

Policy information about studies involving animals; ARRIVE guidelines recommended for reporting animal research, and Sex and Gender in Research

Laboratory animals	C3HeB/FeJ mice were used as a persistent infection model for Mycobacterium abscessus.
Wild animals	N/A
Reporting on sex	This information has not been collected.
Field-collected samples	The study did not involve samples collected from the field.
Ethics oversight	All procedures involving mice were performed in compliance with ethical guidelines and approved by the ethical committee (N°047, agreement A783223) under APAFIS #44601-2023090514417206 v3.

Note that full information on the approval of the study protocol must also be provided in the manuscript.

Plants

Seed stocks	N/A
Novel plant genotypes	N/A
Authentication	N/A
